# Environmental “knees” and “wiggles” as strong stabilizers of species’ range limits set by interspecific competition

**DOI:** 10.1371/journal.pcbi.1014336

**Published:** 2026-06-15

**Authors:** Farshad Shirani, Benjamin G. Freeman

**Affiliations:** 1 School of Mathematics, Georgia Institute of Technology, Atlanta, Georgia, United States of America; 2 Department of Physics, Emory University, Atlanta, Georgia, United States of America; 3 Department of Mathematics and Statistics, Georgetown University, Washington, District of Columbia, United States of America; 4 School of Biological Sciences, Georgia Institute of Technology, Atlanta, Georgia, United States of America; University of Sussex, UNITED KINGDOM OF GREAT BRITAIN AND NORTHERN IRELAND

## Abstract

Whether interspecific competition is a major contributing factor to setting species’ range limits has been debated for a long time. Theoretical studies have proposed that the interactions between interspecific competition and disruptive gene flow along an environmental gradient can halt range expansion of ecologically similar species where they meet. However, the stability of such range limits has not been well addressed. We use a deterministic mathematical model of adaptive range evolution over a continuous habitat to show that the range limits set by interspecific competition are unlikely to be evolutionarily stable if the environmental optima for fitness-related traits vary (almost) linearly in space. That is, in a linear environment without a dispersal barrier or a third (or more) species, the range borders formed between two competing species constantly move towards the weaker species. We demonstrate that environmental nonlinearities such as “knees” and “wiggles”—wherein an isolated sharp change or a step-like change occurs in the steepness of a trait optimum—can strongly stabilize competitively formed range limits. The stabilization mechanism relies on the contrast that such nonlinearities create in the level of disruptive gene flow to the peripheral population of each species, and succeeds when an additional process, such as Allee effects, prevents the establishment of an infinitesimal population in the presence of an abundant competitor. We show that the stability of the range limits at these nonlinearities is robust against moderate environmental disturbances. Whether strong disturbances such as rapid high-amplitude climate changes can destabilize such range limits depends on how the competitive dominance of the species changes across the nonlinearity. Therefore, our findings underscore the importance of assessing species’ competitive ability when predicting responses to climate change, and identify geographic regions where established range limits are likely to persist as well as regions where shifting limits may eventually stabilize.

## Author summary

Individuals of natural populations often compete for resources and space. At the same time, most species have a remarkable capability to adapt to new environments and expand their range. Yet there are no species known to exist globally on the planet. This gives rise to a fundamental question in ecology: what are the factors that limit a species’ range? Interspecific competition has been considered as a major contributor for a long time. However, when the environmental conditions that affect species’ fitness vary linearly, the intrinsic advantage that one species has over the other causes the range limits formed between them to constantly move towards the weaker species. In the absence of a third or more interacting species or a physical barrier, the weaker species either becomes entirely excluded from the habitat or only marginally coexists with the stronger species at the vicinity of the habitat boundary. In the present work, we use a mathematical model of range evolution for two competing species to show that certain nonlinearities in the environmental conditions that determine the species’ performance can strongly stabilize the border formed between the two species due to competition. Such nonlinearities correspond to geographic locations where a sharp change occurs in the steepness of the otherwise linearly changing environment. The moving border between the species eventually stops at the location of the nonlinearities and does not move even when climate changes moderately. With the current concerns about the impacts of climate change on species’ range, our results help predict geographic locations where shifting range limits will eventually settle and and remain fairly insensitive to climate change.

## 1. Introduction

Why are species’ range limits where they are? Understanding the reasons has been a longstanding challenge in ecology [1–8]. Developing such an understanding helps address a fundamental question about the future of biodiversity on the planet: where will species’ range limits be in the near and far future? It is important, for instance, to know whether the currently contracting or expanding range of a species will eventually reach an evolutionarily stable equilibrium state. This equilibrium could correspond to the species’ complete exclusion from the habitat, its full expansion over the entire available habitat, or its existence with a limited range. In the latter case, it is important to foresee where the species’ equilibrium range limits will be, whether they return to the same equilibrium locations after being perturbed by changes in climate, or if they will be destabilized by particular climate changes. Using a mathematical model, we computationally address some of these questions for the case where range limits are formed by interspecific competition and are stabilized by certain environmental nonlinearities.

Since Darwin, ecologists have debated the role of interspecific competition in setting species’ range limits [9–13]. Empiricists have used experiments and comparisons to test whether competition can limit species’ ranges [14–17], while theoreticians have developed mathematical models to explore the conditions in which interspecific competition can set range limits [18–24]. The models used in these theoretical studies may or may not incorporate adaptive evolution, movements across space, and environmental heterogeneity. Non-evolutionary models often include additional factors for setting range limits, such as anti-correlated spatial profiles for species’ carrying capacity, or abrupt shifts in species’ dominance based on their resource utilization dynamics [21,23]. However, it can be argued that, when two species are similar enough to undergo strong competition, they likely have similar resource utilization dynamics and respond similarly to abiotic environments [21]. Further, non-evolutionary models cannot address the possibility that a species may evolve to release itself from the constraining impacts of the other species. Therefore, these models cannot verify whether or not established range limits will be evolutionarily stable [23].

Among studies that consider adaptive evolution, the work of Price and Kirkpatrick [23] proposes that interspecific competition can set evolutionarily stable range limits even in the absence of disruptive effects of gene flow, and even in the case where species are competitively unequal. However, their analysis does not include species’ movement. As a result, the possibility remains that the established range limits constantly move in space due to the difference between competition strengths of the species. This means that the limits will not be evolutionarily stable. In fact, non-spatial models and models that do not incorporate movements implicitly assume that there exist external forces or internal physiological limitations on species’ individuals which prevents them from moving to other locations. Given that even non-mobile species such as plants can sometimes disperse long distances, these assumptions are unlikely to hold for most real-world cases. The presence of dispersal-preventing forces in natural organisms is rare. Further, studying range evolution of species with restricted dispersal or movement capabilities is of less importance in understanding causes of species’ range limits.

In a seminal work incorporating species movement, Case and Taper [20] used a comprehensive evolutionary model to demonstrate that the interaction between interspecific competition and gene flow along environmental gradients can limit species’ range expansion. However, the stability of the range limits in their two-species simulations was established for the non-generic case of identical competitors, which is unlikely to ever be the case in nature. Moreover, intraspecific phenotypic variance in Case and Taper’s model [20] is constant over space and time, an assumption they describe as “tenuous”. Relaxing this assumption for a single (solitary) species results in different conclusions [24,25], weakening confidence in the results presented by Case and Taper [20]. The main finding of Case and Taper’s influential work, that the interaction between interspecific competition and gene flow can set range limits, has been reaffirmed by Shirani and Miller [24] for the case where species’ phenotypic variance is free to evolve. However, it still remains unclear if interspecific competition can set evolutionarily stable range limits between two species in the general case of unequal competitors.

In the present work, we aim to investigate the stability of the range limits formed by interspecific competition and identify effective environmental stabilizers of such range limits. To accomplish our goals, we use the mathematical model of species’ range evolution developed by Shirani and Miller [24], an extension of Case and Taper’s model [20] that allows for the evolution of species’ trait variance in space and time. Using a system of deterministic partial differential equations (PDEs), the model presents the joint evolution of the population density and the intraspecific mean and variance of a quantitative phenotypic trait for a community of competitively interacting species. We further extend the model to additionally incorporate Allee effects on species’ population growth. The presence of a process such as Allee effect or genetic drift—that prevents infinitesimal populations of a competitively stronger species from establishing themselves within the range of a weaker species—is necessary (but not sufficient) for the stability of range limits formed by interspecific competition. We draw our conclusions by numerically computing the solutions of the model in different eco-evolutionary regimes.

We first show that the range limits set by interspecific competition between two species are unlikely to be evolutionary stable in a linear (or almost linear) environment (see Box 1). We then propose and analyze a reasonable stabilizing factor for the range limits: environmental nonlinearities in the form of “knees” and “wiggles” (see Box 1). The latter is also studied in [20] for the special case of identical competitors. These nonlinearities appear as relatively sharp changes in the steepness of the spatial profile of the environmental optimum for a fitness-related quantitative trait. Environmental wiggles and knees are likely prevalent in the real world, as nonlinear spatial changes in climate, geology, habitat structure, or biotic communities are commonly observed along environmental gradients. We show that these nonlinearities can “strongly” (see Box 2) stabilize competitively formed range limits. We further show that the established stability at the nonlinearities is fairly robust against environmental disturbances caused by climate change, and that the competitive dominance of the competing species across the nonlinearities is important for predicting range shifts in response to climate change.

**Box 1. Ecological and evolutionary terminology.**

**Environmental gradients:** In general, an *environmental gradient* along a geographic space refers to a spatial change in the environmental conditions that affect species’ fitness and performance. Such changes in environmental factors can cause, for example, a gradient in species’ productivity which can be modeled as spatial changes in the carrying capacity of the environment. Environmental conditions such as temperature, humidity, light density, and resource quality can also cause gradients in the most suitable (optimum) phenotypes. We say an environment is *linear* if the effects of all environmental conditions on the fitness (growth rate) of the species vary linearly in space, that is, with constant gradient. Throughout the present work, an environmental gradient always refers to a selective gradient that the environment imposes on the individuals by setting the optimum value of a fitness-related quantitative trait (phenotype). Therefore, the environment is linear if the trait optimum changes linearly in space. Other environment-dependent parameters are assumed to be constant in space. However, our results remain qualitatively valid if changes in such factors occur linearly.

**Environmental knees:** By *environmental knee* we refer to a point along an environmental gradient in trait optimum at which the steepness of the gradient changes sharply; see the black line in Fig 2b.

**Environmental wiggles:** By *environmental wiggle* we refer to a step-like nonlinearity in the environmental trait optimum, across which the steepness of the gradient changes sharply from an initially moderate value to a significantly higher value, and then back to a moderate value; see the black line in Fig 2d. This means that, outside a wiggle the gradient is moderate/shallow, whereas inside the wiggle it is steep. A wiggle is therefore composed of two adjacent knees, with opposite directions of changes in the gradient steepness. We further require the spatial span of the nonlinearity to be relatively short; approximately shorter than ten times the mean dispersal distance of the individuals in one generation time. Exceedingly wide wiggles are better understood as two separate knees. We refer to the geographic region within a wiggle (i.e., between the two knees) as the *transition zone* of the wiggle, and to the steepness of the environmental gradient over the transition zone as the *steepness* of the wiggle.

**Fitness loads:** In general, we refer to any factor that causes a reduction in the fitness (growth rate) of individuals, or the mean fitness of populations, as a *fitness load*. Depending on the nature of the factor and the fitness component it affects, we may further specify it as a *migration load* (caused by random gene flow), *genetic/phenotypic load* (caused, for example, by natural selection), *competition load*, or *genetic drift load*.

**Phenotype utilization distributions:** The *phenotype utilization distribution* (curve) of an individual with phenotype *p* is a probability density function, which we denote by $\psi_{p}$. When evaluated at a phenotype value $\tilde{p}$, $\psi_{p}$($\tilde{p}$) gives the probability density that an individual with phenotype *p* will utilize a unit of resource that is most favorable for (i.e., is expected to be mostly utilized by) an individual with phenotype $\tilde{p}$. Assuming that the environmental resources vary continuously along a resource axis, and that there exists a one-to-one mapping between the resource values and phenotype values, the phenotype utilization curves are computed based on resource utilization curves through identifying the resource axis by the phenotype axis [18, Eq. (24.5)]. See Appendix A.2 and Section 3.2 in [24] for further details.

## 2. Materials and methods

Our work is based on a deterministic mathematical model of eco-evolutionary range dynamics in a community of competing species that disperse diffusively (randomly) in a heterogeneous environment. The model, developed by Shirani and Miller [24] as an extension of the seminal works of Kirkpatrick and Barton [26] and Case and Taper [20], has a quantitative phenotypic framework. Species’ adaptation and evolution occur based on the value of a phenotypic trait possessed by each individual in the community. The trait is assumed to be strongly correlated with individuals’ fitness. The model incorporates the effects of directional and stabilizing natural selection, which act to reduce the density of the phenotypes that differ from an optimum phenotype imposed by the environment. Intra- and inter-specific competition between the species’ individuals are phenotype-dependent. Populations are assumed to be fully polymorphic. All phenotypes are assumed to be present in each population at all times, but with different frequencies. Evolution proceeds by the differential growth of phenotypes under the selective force of the frequency-dependent competition, as well as the genetic load imposed by natural selection. Phenotypic variability is assumed to be predominantly genetic, and genetic variation is assumed to be predominantly additive. That is, $H^{2}\approx h^{2}\approx 1$, where H^2^ and h^2^ denote broad- and narrow-sense heritability, respectively. We also include Allee effects on population growth to prevent infinitesimal populations from unrealistically disturbing formation and stability of the range limits; see the Results section for more details.

We obtain our results by numerically solving the equations of the model for two ecologically similar species, one of them being competitively stronger than the other, living along a one-dimensional geographic region (habitat). We use the same numerical method as used in [24] to solve the equations, with slight modifications. The details of the numerical scheme and technical challenges in computing the solutions are provided in Appendix C in S1 Appendix. We model an environmental knee by sharply changing the steepness of the environmental gradient (in the trait optimum) at a given geographic point, and an environmental wiggle by making a step-like change in the trait optimum. We analyze the evolution and stability of the species’ competitively formed range limits as they enter and get stabilized in an environmental knee or wiggle. We test the robustness of the stability of the range limits against moderate and strong climate-warming disturbances.

It is important to note that our numerical simulation-based analyses cannot serve as mathematical proofs of stability of the range limits. A rigorous mathematical analysis of the stability of the range limits in the PDE model that we use, in an infinite-dimensional dynamical systems framework, is quite challenging and is beyond the scope of this work. In the results we present, the coevolutionary mechanisms of range stabilization that we describe in words are the main arguments that convince us of the stability (and its type) of the range limits formed at environmental knees and wiggles. Our numerical simulations serve to demonstrate and support our verbal descriptions.

Before presenting the results, we describe the essential components of the model governing the underlying dynamics of the species’ range evolution and provide the complete set of model equations. We present the formulations for a reduced version of Shirani and Miller’s model [24] for two interacting species in a one-dimensional geographic space. We refer the reader to their work for further details of the model for multiple species in higher dimensional spaces. Our new addition of Allee effects slightly changes the formulations of the equations presented in [24].

### 2.1. Model Components

We describe the model for adaptive range evolution of two competing species in a one-dimensional habitat Ω=(a,b)⊂ℝ over an evolution time horizon from *t* = 0 to *t* = *T* > 0. The definitions of *t*he parameters used for presen*t*ing the model are given in Table 1, along with their default values used in our simulations. At every location x∈Ω and time t∈[0,T], the *i*th species’ range dynamics is governed by three coupled equations that control the joint evolution of the population density $n_{i}(x,t)$, intraspecific trait mean $q_{i}(x,t)$, and intraspecific trait variance $v_{i}(x,t)$, where *i* = 1,2. These main equations of the model are given as Eqs. (8)–(10) in next section. The basic contributing components of the model are described as follows.

**Table 1:** Definition and default values of the parameters of the model (5)–(7). The default values are used in the simulations presented in the Results section, unless otherwise is stated.

Parameter	Definition	Default	Unit
D_*i*_	Diffusion coefficient of the *i*th species’ dispersal	1	${𝚇}^{2}/𝚃$
K_*i*_	Carrying capacity of the environment for *i*th species^*^	1	𝙽/𝚇
J_*i*_	Critical population density (Allee threshold) of *i*th species	0.02	𝙽/𝚇
R_*i*_	Maximum per capita growth rate of *i*th species	1	1/𝚃
V_*i*_	Variance of individuals’ phenotype utilization in *i*th species	9	${𝚀}^{2}$
S	Measure of the strength of natural selection	0.2	${𝚀}^{-2}/𝚃$
U	Rate of increase in trait variance due to mutation	0.02	${𝚀}^{2}/𝚃$
Q(*x*)	Optimal trait value for the environment	Linear^†^	𝚀
$\left|\partial_{x}Q(x)\right|$	Steepness (magnitude) of the gradient of the optimal trait	0.5	𝚀/𝚇

^*^The carrying capacity K_*i*_ denotes the maximum population density *n*_*i*_ that the *i*th specie can attain, when its individuals are “completely generalist”, that is, when $V_{i}\to \infty$. Small values of *V*_*i*_ release the populations from strong competition, letting *n*_*i*_ exceed K_*i*_.

^†^The default value “Linear” specified for Q means that Q is by default considered to be a linear function of *x* over Ω.

Before proceeding to present the equations, we note that the mathematical formulations given in [24] have some notational differences compared with the preceding foundational models [20,26], which we preserve here. To facilitate comparisons with preceding models, we provide a list of notational differences with [20] in Table 2. In Appendix A in S1 Appendix, we also provide the complete set of model equations rewritten based on the notations used in [20].

**Table 2:** Notational comparison between the model presented in this work and Case & Taper’s model [20]. The presence of the enumeration index *i* in a variable implies that the variable can take a different value for each species.

Parameter/variable/function description	Present work	Case & Taper’s [20]
Trait (phenotype) values	*p*	*z*
Population density	*n*_*i*_	*N*_*i*_
Trait mean	*q*_*i*_	${\bar{z}}_{i}$
Trait variance	*v*_*i*_	*V*_*p*_
Per capita intrinsic growth rate	*g*_*i*_	*w*_*i*_
Mean intrinsic growth rate	*G*_*i*_	${\bar{w}}_{i}$
Competition kernel	$\alpha_{ij}$	α
Relative frequency (distribution) of phenotypes	$\phi_{i}$	*p*_*i*_
Mutational changes in frequency of phenotypes	$\partial_{t}^{\left(M\right)}\phi_{i}$	—
Diffusion coefficient of species’ dispersal	D_*i*_	*D*
Carrying capacity of the environment	K_*i*_	*K*
Critical population density (Allee threshold)	J_*i*_	—
Maximum per capita growth rate	R_*i*_	*r*_*i*_
Variance of individuals’ phenotype utilization	V_*i*_	*V*_u_
Measure of the strength of natural selection	S	$1/V_{s}$
Rate of increase in trait variance due to mutation	U	—
Optimal trait value for the environment	Q	θ
Steepness of the gradient of the optimal trait	$\left|\partial_{x}Q(x)\right|$	*b* (for linear θ)
Competition variance (Eq. (12) in Case & Taper)	$\frac{1}{2}(v_{i}+v_{j}+2{\bar{V}}_{ij})$	$V_{\alpha }^{*}:=V_{p}+V_{u}$

Let $\phi_{i}(x,t,p)$ denote the relative frequency of phenotype value p∈ℝ within the *i*th species’ population. Then $n_{i}(x,t)\phi_{i}(x,t,p)$ gives the population density of individuals with phenotype *p* in the *i*th population. The model assumes that changes in $n_{i}(x,t)\phi_{i}(x,t,p)$ over a small time interval of length τ→0 results from the contribution of three major factors, as:


$$\begin{array}{cc} n_{i}(x,t+\tau )\phi_{i}(x,t+\tau ,p)- & \;n_{i}(x,t)\phi_{i}(x,t,p)\\ = & \;\tau D_{i}\;\partial_{x}^{2}(n_{i}(x,t)\phi_{i}(x,t,p)) \end{array}$$ (1a)

$$\begin{array}{c} +\tau g_{i}(x,t,p)n_{i}(x,t)\phi_{i}(x,t,p) \end{array}$$ (1b)

$$\begin{array}{c} +\tau n_{i}(x,t)\partial_{t}^{\left(M\right)}\phi_{i}(x,t,p), \end{array}$$ (1c)

where $\partial_{x}^{2}$ denotes the second partial derivative with respect to *x*. The first contributing factor (1a) represents the diffusive (random) dispersal of individuals to and from neighboring locations, where D_*i*_ denotes the diffusion coefficient; see Table 1. The second contribution (1b) is due to the intrinsic growth of the individuals with phenotype *p* in the *i*th population, the rate of which is denoted by $g_{i}(x,t,p)$. Finally, the third factor (1c) represents mutational changes in the relative frequency of the phenotype *p*, occurring at a rate denoted by $\partial_{t}^{\left(M\right)}\phi_{i}(x,t,p)$. The probability of mutational changes from a phenotype *p* to another phenotype $p^{\prime }$ is assumed to be dependent on the difference $\delta p=p-p^{\prime }$ between the phenotypes. Letting ν(δp) denote the probability density of the occurrence of such mutational changes, the model further assumes that ν follows a distribution with zero mean and constant variance. Based on these assumptions, the formulation used in the model for $\partial_{t}^{\left(M\right)}\phi_{i}(x,t,p)$ is given in [24, Section A.3].

The key factor that differentiates the density of individuals with different phenotypes is their intrinsic growth rate, which is modeled as,


$$\begin{array}{c} g_{i}(x,t,p):=R_{i}\left(1-\frac{1}{K_{i}}\sum_{j=1}^{2}n_{j}(x,t)\int_{\mathbb{R}}\alpha_{ij}(p,p^{\prime })\phi_{j}(x,t,p^{\prime })dp^{\prime }\right)B_{i}(n_{i}(x,t)) \end{array}$$ (4)

$$\begin{array}{c} \;-\frac{S}{2}(p-Q(x){)}^{2}, \end{array}$$ (5)

where S denotes the strength of stabilizing selection, Q(*x*) denotes the environment’s optimal trait value at location *x*, R_*i*_ denotes the maximum per capita growth rate of the *i*th species, and K_*i*_ denotes the carrying capacity of the environment for the *i*th species when the species’ individuals are completely generalist; see Table 1 and the clarification note below. The rest of the terms are described below. Note that, in writing Eq. (4), it is essentially assumed that the reproduction rate of the individuals with phenotype *p* follows a logistic growth (with Allee effect), with R_*i*_ and K_*i*_ being independent of *p*.

The convolution term in Eq. (4) models the effects of phenotypic competition between the individuals. Specifically, the competition kernel $\alpha_{ij}(p,p^{\prime })$ denotes the strength of per capita effects of individuals with phenotype $p^{\prime }$ in the *j*th species on the frequency of individuals with phenotype *p* in the *i*th species. This competition kernel is specified by the MacArthur-Levins overlap [27] between *phenotype utilization curves* of the individuals (see Box 1), assuming that the environmental resources vary continuously along a resource axis. The phenotype utilization distribution (curve) associated with individuals with phenotype *p* in the *i*th population is assumed to be normal, with mean *p* and constant utilization variance V_*i*_. The overlap between these curves gives the competition kernel


$$\begin{array}{c} \alpha_{ij}(p,p^{\prime })=\sqrt{V_{i}/{\bar{V}}_{ij}}\exp\left(-\frac{(p-p^{\prime }{)}^{2}}{4{\bar{V}}_{ij}}\right),\;i,j\in \{1,2\}, \end{array}$$ (6)

where ${\bar{V}}_{ij}:=\frac{1}{2}(V_{i}+V_{j})$. The details are given in [24, Sections A.2 and A.4].

Before proceeding to define the remaining terms in Eq. (4) and (5), it is important to clarify an ambiguity in the definition of K_*i*_ that is present due to the phenotype-dependence of competition in the model. As stated above, K_*i*_ denotes the maximum population density of the *i*th species when the species’ individuals are “completely generalist”, that is, when $V_{i}\to \infty$, *i* = 1,2. This is because $\alpha_{ij}(p,p^{\prime })\to 1$ in Eq. (6) when ${\bar{V}}_{ij}\to \infty$, regardless of the values of *p* and $p^{\prime }$. Setting $\alpha_{ij}(p,p^{\prime })=1$ in Eq. (4) and (5) verifies that completely generalist species with density *n*_*i*_ > K_*i*_ will have negative intrinsic growth. However, when individuals are specialized in utilizing resources, they become partially released from competition, meaning that $\alpha_{ij}(p,p^{\prime })<1$. In this case, *g*_*i*_ can take positive values for some *n*_*i*_ > K_*i*_. That is, specialized species can grow to population densities greater than K_*i*_. For example, see the curves of range expansion wave amplitudes (maximum population density) given in [28, Fig 2b]. Therefore, even though K_*i*_ is phenotype-independent, the carrying capacity of species—defined generally as species’ maximum population density—depends both on the distribution of phenotypes and individuals’ phenotype-based resource utilization. Unless explicitly specified as K_*i*_, in our references to carrying capacity in the rest of this paper we always mean populations’ maximum attainable density.

The nonlinear function *B*_*i*_ of the species’ population density in Eq. (4) incorporates the Allee effect. We assume that, due to the Allee effect, a population whose density is below a critical density, the *Allee threshold*, will have a negative intrinsic growth rate. We denote the critical density for the *i*th species with J_*i*_. When the population’s density increases above the critical density, the population’s intrinsic growth rate increases sharply. For a fully adapted solitary population with completely generalist individuals, the increase in growth rate with density continues until the the maximum growth rate R_*i*_ is attained. Further increases in the density will then result in a gradual decrease in the growth rate, eventually to zero when the density reaches the carrying capacity. For this, we define *B*_*i*_ as


$$\begin{array}{c} B_{i}(n_{i}):=B_{i}\left(\frac{1}{1+\exp\left(-\frac{n_{i}-J_{i}}{\sigma_{i}}\right)}-\frac{1}{2}\right), \end{array}$$ (7)

where $\sigma_{i}$ is a parameter for adjusting the sharpness of the growth rate increase to its maximum, and B_*i*_ is a parameter for adjusting the maximum growth rate (to be R_*i*_). For both species throughout our study, we set $J_{i}=0.02\;𝙽/𝚇$, $\sigma_{i}=0.05\;𝙽/𝚇$, and $B_{i}=2.6$. The resulting profile of the intrinsic growth rate (2) is shown in Fig A in S1 File.

Finally, the quadratic term (2b) in the intrinsic growth function imposes directional and stabilizing forces of natural selection on individuals with phenotype *p*. It penalizes phenotypes that are far from the environmental optimal phenotype Q, thereby facilitating population adaptation to new environments. In our results, we model environmental wiggles by inserting short-range step-like nonlinearities in the otherwise linear profile of Q.

The phenotype density evolution Equations (1–3), along with the definition of its components as described above, is used to derive the final equations of the model for the evolution of *n*_*i*_, *q*_*i*_, and *v*_*i*_, as given in Eqs. (8)–(10) below. The Allee effect term $B_{i}(n_{i})$ that we have included in Eq. (4) and (5) does not depend on the phenotype value *p*. As a result, following the derivations provided in [24] simply yields a replacement of R_*i*_ with $B_{i}(n_{i})R_{i}$ in the final equations derived in [24]. We note that the master Equation (1–3) is specified at the phenotypic level. Therefore, the derivation of the equations in [24] is performed entirely at the phenotypic level. The derivations rely on a key assumption, inherited from the preceding foundational models [20,26], that phenotype values in each species are normally distributed at every occupied habitat location for all time. This assumption allows for exact moment closure in deriving the equations for trait means (by computing first moment of phenotypes) and trait variances (by computing the second moment of phenotypes). See Appendix B in S1 Appendix for a discussion on the plausibility of this assumption in our study. As stated above, in deriving the equations of trait variance, it is also implicitly assumed in [24] that phenotypic variation is predominantly caused by additive genetic variation ($H^{2}\approx h^{2}\approx 1$).

### 2.2. Model equations

The final equations of the model for two competing species are presented by a system of partial differential equations that governs the evolution of each species’ population density $n_{i}(x,t)$, *i* = 1,2, trait mean $q_{i}(x,t)$, and trait variance $v_{i}(x,t)$ at every habitat location x∈Ω and time t∈[0,T], *T* > 0. For brevity, let $u:=(n_{1},q_{1},v_{1},n_{2},q_{2},v_{2})$ be a vector containing all model variables. Moreover, let the partial derivatives with respect to *t* and *x* be denoted by $\partial_{t}$ and $\partial_{x}$, respectively, and the second partial derivative with respect to *x* be denoted by $\partial_{x}^{2}$. Then, the equation for the evolution of the population density *n*_*i*_ of the *i*th species is given by


$$\begin{array}{c} \partial_{t}n_{i}(x,t)=D_{i}\partial_{x}^{2}n_{i}(x,t)+G_{i}(x,u(x,t))n_{i}(x,t), \end{array}$$ (8)

where $G_{i}(x,u)$ denotes the mean growth rate of the population as given by Eq. (11) below. Likewise, equations for the evolution of the trait mean *q*_*i*_ and the trait variance *v*_*i*_ within the *i*th species are given as


$$\begin{array}{c} \partial_{t}q_{i}(x,t)=D_{i}\partial_{x}^{2}q_{i}(x,t)+2\;D_{i}\partial_{x}(\log n_{i}(x,t))\partial_{x}q_{i}(x,t)+H_{i}(x,u(x,t)), \end{array}$$ (9)

and


$$\begin{array}{cc} \partial_{t}v_{i}(x,t) & =D_{i}\partial_{x}^{2}v_{i}(x,t)+2\;D_{i}\partial_{x}(\log n_{i}(x,t))\partial_{x}v_{i}(x,t)\\ & \;+2\;D_{i}(\partial_{x}q_{i}(x,t){)}^{2}+W_{i}(x,u(x,t)). \end{array}$$ (10)

The nonlinear mappings *G*_*i*_, *H*_*i*_, and *W*_*i*_ used in Eqs. (8)–(10) are defined as


$$\begin{array}{c} G_{i}(x,u)=B_{i}(n_{i})\left(R_{i}-\frac{R_{i}}{K_{i}}\sum_{j=1}^{2}M_{ij}(u)C_{ij}(u)n_{j}\right)-\frac{S}{2}\left((q_{i}-Q(x){)}^{2}+v_{i}\right), \end{array}$$ (11)

$$\begin{array}{c} H_{i}(x,u)=(B_{i}(n_{i})R_{i}-G_{i}(x,u))q_{i}-B_{i}(n_{i})\frac{R_{i}}{K_{i}}\sum_{j=1}^{2}L_{ij}(u)M_{ij}(u)C_{ij}(u)n_{j}+E_{i}(x,u), \end{array}$$ (12)

$$\begin{array}{c} W_{i}(x,u)=(B_{i}(n_{i})R_{i}-G_{i}(x,u))(v_{i}-q_{i}^{2})-B_{i}(n_{i})\frac{R_{i}}{K_{i}}\sum_{j=1}^{2}P_{ij}(u)M_{ij}(u)C_{ij}(u)n_{j}+Y_{i}(x,u), \end{array}$$ (13)

where *B*(*n*_*i*_) is given by Eq. (7) and, letting ${\bar{V}}_{ij}:=\frac{1}{2}(V_{i}+V_{j})$, i,j∈{1,2},


$$\begin{array}{c} C_{ij}(u):=\sqrt{\frac{2V_{i}}{v_{i}+v_{j}+2{\bar{V}}_{ij}}}, \end{array}$$ (14)

$$\begin{array}{c} M_{ij}(u):=\exp\left(-\frac{(q_{i}-q_{j}{)}^{2}}{2(v_{i}+v_{j}+2{\bar{V}}_{ij})}\right), \end{array}$$ (15)

$$\begin{array}{c} L_{ij}(u):=\frac{v_{i}q_{j}+(v_{j}+2{\bar{V}}_{ij})q_{i}}{v_{i}+v_{j}+2{\bar{V}}_{ij}}, \end{array}$$ (16)

$$\begin{array}{c} P_{ij}(u):=\frac{v_{i}(v_{j}+2{\bar{V}}_{ij})}{v_{i}+v_{j}+2{\bar{V}}_{ij}}+L_{ij}(u)(L_{ij}(u)-2q_{i}), \end{array}$$ (17)

$$\begin{array}{c} E_{i}(x,u):=\frac{S}{2}\left[2Q(x)v_{i}+2Q(x)q_{i}^{2}-Q^{2}(x)q_{i}-3v_{i}q_{i}-q_{i}^{3}\right], \end{array}$$ (18)

$$\begin{array}{c} Y_{i}(x,u):=\frac{S}{2}\left[2Q(x)v_{i}q_{i}-2Q(x)q_{i}^{3}-Q^{2}(x)(v_{i}-q_{i}^{2})-3v_{i}^{2}+q_{i}^{4}\right]+U. \end{array}$$ (19)

As stated before, the definitions of the model parameters and their default values used in the simulations are given in Table 1.

Solving the equations of the model (5)–(7) over a habitat Ω=(a,b) requires specifying boundary conditions at habitat boundary points *x* = *a* and *x* = *b*. For the numerical studies presented throughout our work we always assume that there is no phenotype flux through the boundary of the habitat, which gives the homogeneous Neumann boundary conditions


$$\begin{array}{c} \partial_{x}n_{i}=0,\;\partial_{x}q_{i}=0,\;\partial_{x}v_{i}=0,\;i=1,2\;\text{on }\{a,b\}\times [0,T]. \end{array}$$ (20)

Equivalently, these conditions imply that the habitat boundary is reflecting.

### 2.3. Units and parameter values

The choice of units for the physical quantities of the model is an important factor in suggesting biologically reasonable values for model parameters based on available estimates in the literature. Due to the complexity of the equations, analytical studies of the behavior of the model in different evolutionary regimes is rather impractical. Numerical studies of the model, as we perform in this work, will then require carefully chosen parameter values such that the resulting predictions are biologically reasonable. An extensive discussion is provided in [24] on the choice of units and reasonable ranges of parameter values. Below, we describe a summary of that discussion as needed for understanding the values given in Table 1.

To specify units for the physical quantities of the model, one of the species in the community is first chosen as a *representative species*, for example, the one which is best adapted to the environment or has the widest niche. The units are then specified based on the measurements made on this representative species. Specifically, the unit of time, denoted by 𝚃, is set to be equal to the mean generation time of the representative species. The unit of space, denoted by 𝚇, is chosen such that the diffusion coefficient D of the representative population becomes unity. That is, 1𝚇 is set to be equal to the root mean square of the dispersal distance of the population in 1𝚃, divided by $\sqrt{2}$. Having set the unit of space, the unit of measurement for population abundances, 𝙽, is then chosen such that 1𝙽 is equal to the carrying capacity of the environment for 1𝚇 unit of habitat length. Note that this results in the default value of K_*i*_ for the representative population to become unity, provided the individuals of the population are sufficiently generalist. Finally, letting 𝚀 denote the unit of measurement for the quantitative trait, 1𝚀 is set to be equal to one standard deviation of the trait values at the core of the representative population.

As shown in [29, Section 3.2], the strength of natural selection S can be estimated as $S\approx -\gamma +\beta^{2}$, where γ denotes the standardized stabilizing selection gradient and β denotes the standardized directional selection gradient [30–32]. Estimates of these standardized selection gradients are available in the literature, see for example [31,33,34]. These estimates can be directly used to provide an estimate for S in our model, as they are obtained based on one generation (our unit of time) of selection for a trait measured in standard deviation units (our unit of trait). Estimates for the mutational rate of increase in trait variance are available in the literature, either relative to environmental variance or relative to standing genetic variance [35]. Since we choose standard deviation of the trait in the representative population as the unit of trait, the standing genetic variance can be approximated to be unity. Since it is also implicitly assumed in our model that phenotypic variation is almost equal to genetic variation ($H^{2}\approx 1$), the available estimates in [35] can be used directly as approximate estimates for U in the model we use; see [24] for further discussion. Estimates for the variance of phenotype utilization distribution V_*i*_ can be obtained by computing the within-phenotype component of species’ niche breadth, as defined in trait-based niche quantification approaches [24,36,37]. Based on our choices of unit for trait mean, the estimates are expected to be greater than 1, since the local representative population might not yet have filled its entire possible niche.

We use a toy example to illustrate how parameter values measured in arbitrary units are converted to values based on our chosen units. Consider a species of bird whose wing length (trait) increases as the partial pressure of oxygen decreases across an elevational gradient. Suppose a well-adapted representative local population of this species has a trait distribution with standard deviation equal to 5 mm. Suppose further that the environmental optimum for the trait increases linearly across the habitat, with a slope equal to 2 mm/km. The individuals are generalist, and the carrying capacity of the environment is equal to 4000 individuals per kilometer. Note that, for simplicity, we assume that the habitat is one-dimensional. The maximum per capita intrinsic growth rate of the individuals is 0.3 per year. The mean generation time of the species is 3.5 years and its mean dispersal distance is 0.7 kilometer per year. An estimate for individuals’ phenotype utilization variance obtained based on trait-based niche quantification is equal to 100 mm^2^. Estimates for the standardized directional and stabilizing selection are β=0.2 and γ=−0.15, based on one generation of selection and for wing lengths measured in standard deviation units. For this example species, we compute the parameter values based on our choices of units as follows.

The unit of time is set equal to the mean generation time, 1𝚃=3.5years. The mean dispersal distance per generation is equal to 0.7×3.5=2.45 kilometers. Therefore, we set the unit of space as $1\;𝚇=2.45/\sqrt{2}\approx 1.73\;\text{km}$. The carrying capacity for one unit of habitat length is then 4000×1.73=6920 individuals. We set the unit of population abundance as 1N=6920 individuals. Finally, we set the standard deviation unit for trait, that is 1𝚀=5mm. Based on these units, it follows immediately that $D=1\;{𝚇}^{2}/𝚃$, and K=1𝙽/𝚇. The maximum intrinsic growth rate is $R=0.3\times 3.5=1.05\;{𝚃}^{-1}$. The phenotype utilization variance is $V=100/5^{2}=4\;{𝚀}^{2}$. The strength of selection is $S\approx -\gamma +\beta^{2}=0.15+0.2^{2}=0.19\;{𝚀}^{-2}/𝚃$. Finally, the slope of the environmental gradient is computed as 2×1.73=3.46mm/𝚇, which gives the slope $\partial_{x}Q=3.46/5\approx 0.7\;𝚀/𝚇$ based on our units. The estimates in [24] suggest that this environmental gradient is quite steep.

## 3. Results

We investigate how the interaction between interspecific competition and gene flow leads to formation of range limits between two competing species, and how the limits are stabilized by environmental knees and wiggles. For this, we numerically solve the equations of the model (5)–(7) given above, with their nonlinear terms given by Eqs. (11)–(19). We perform our simulations in the one-dimensional habitat Ω=(0𝚇,50𝚇)⊂ℝ with the reflecting boundary conditions (17). We set the spatial profile of the trait optimum Q to be decreasing along the habitat. This simplified habitat can be conceptualized to represent, for example, the living environment of two species of montane birds over an elevation-dependent temperature gradient, with Q representing a temperature-correlated phenotypic trait (for example, clutch size is a trait in birds which decreases with elevation [38]). The reflecting boundary condition at *x* = 50 can then be equivalent to symmetrically (reflectively) expanding the habitat at the mountain top. Our choice of the default value $R_{i}=1\;{𝚃}^{-1}$ in Table 1 is also purposed to agree with a typical value of the maximum growth rate per generation for birds [24,39,40].

In all simulations, we initially introduce the two populations allopatrically, considering the 1st population as the *downslope* (at lower elevations) and the 2nd population as the *upslope* species. We set the initial population densities at t=0𝚃 as $n(x,0)=0.5\varrho ((x-c_{i})/2)$, where *c*_*i*_ denotes the center of the *i*th population and ϱ is a bump function with support [−1, 1]; see also Appendix C in S1 Appendix. We assume that the initial populations are perfectly adapted to their environment at their center, that is, $q_{i}(c_{i},0)=Q(c_{i})$. However, we assume fairly poor initial adaptation at other habitat locations by letting the slope of the initial trait means vary as $\partial_{x}q_{i}(x,0)=0.1\;\partial_{x}Q(x)$, for all x∈Ω. Finally, we assume that the initial populations have a constant trait variance of $v_{i}(x,0)=1\;{𝚀}^{2}$.

### 3.1. Maladaptive effects of random gene flow

The evolutionary mechanism of range limits stabilization that we describe in the present work relies on the maladaptive effects of gene flow created by random dispersal of individuals. Understanding these effects is important for better understanding the range evolution processes we present. Therefore, before proceeding to the main results, we analyze the range evolution of a single (solitary) species to show how the maladaptive effects of gene flow affect the range expansion dynamics, and to clarify the distinction between these effects and the contrasting effects of gene flow that increase adaptive potential of species. For this, we solve the equations of a single-species version of the model (Eqs. (A17)–(A19) in S1 Appendix) in a linear environment. We use the solutions to quantify, separately, the effects of dispersal and local effects of selection, competition, and genetic variation on rate of change of trait mean $\partial_{t}q$ (adaptation/maladaptation rate). To avoid interrupting the flow in presenting the main results, we provide this detailed analysis in Appendix D in S1 Appendix. Here, we only use the results to clarify our reference to maladaptive effetcs of gene flow, as follows.

As an initially introduced population gradually adapts to new environments and expands its range (Fig A1a in S1 Appendix), the trait mean at the core of the population converges to the environmental trait optimum (Fig A1b in S1 Appendix). Since the population’s density declines near the range edge, the gene flow created by random dispersal is asymmetric, that is, predominantly from the abundant core to the scarce edge. Due to the presence of a gradient in the trait optimum, the phenotypes that are adapted to the environment at the core will be maladapted near the edge. Therefore, this asymmetric gene flow reduces the mean population fitness (growth rate) at range margins and slows down range expansion. The steeper the environmental gradient, the stronger the maladaptive effects of gene flow and the slower the range expansion speed.

As in [26], throughout the present work, by “maladaptive”, “disruptive”, or “swamping” effects of asymmetric core-to-edge gene flow we refer to the deviation of trait mean from trait optimum at peripheral populations due to receiving maladapted phenotypes from the core (often known as migration load). These maladaptive effects are quantified in Appendix D in S1 Appendix and shown in Figs A1d, A2a, and A2d in S1 Appendix at different levels of environmental gradient steepness. In the results presented in next sections we show how these maladaptive effects of gene flow are intensified by the interactions with interspecific competition, leading to formation of range limits, as in [20,24]. We also show how these effects play the key role in stabilizing range limits at environmental knees and wiggles.

### 3.2. Interspecific competition, asymmetric gene flow, and formation of species’ range limits

**Fig 1:**
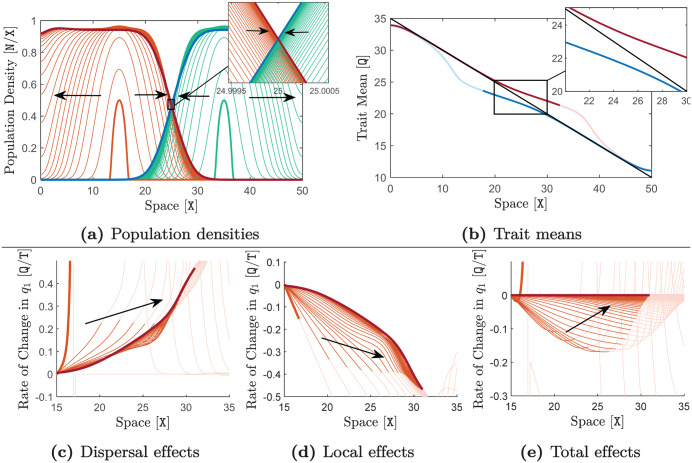
Evolution of range limits, region of sympatric coexistence, and character displacement for two competitively identical species. Both species have the same parameter values equal to the default values given in Table 1. The trait optimum Q is linear and decreasing, shown by the black line in **(b)**, with a moderately steep gradient of $\partial_{x}Q=-0.5\;𝚀/𝚇$. Curves of the species’ population density are shown in **(a)**. The final curves of the species’ trait mean obtained at the end of the simulation are shown in **(b)**. The contribution of dispersal (gene flow) to the rate of change of the 1st species’ trait mean $\partial_{t}q_{1}$ is shown in **(c)**. The local contributions of selection and competition to $\partial_{t}q_{1}$ are shown in **(d)**. The curves are shown only for the right-half of the 1st species’ range, (15𝚇,50𝚇). Curves in (c) are computed using the term $D_{i}\overset{2}{\underset{x}{\partial }}q_{i}+2D_{i}\partial_{x}(\log n_{i})\partial_{x}q_{i}$ in Eq. (9), with *i* = 1. Curves in (d) are computed using the term *H*_*i*_ in Eq. (9), with *i* = 1. The total contribution to $\partial_{t}q_{1}$, shown in **(e)**, is computed as the sum of the curves shown in (c) and **(d)**. For the downslope (left) species, curves are shown in orange, thick orange curves indicate the initial curves at t=0𝚃, and the final curves at the end of the simulation are highlighted in red. For the upslope (right) species, curves are shown in green, thick green curves indicate the initial curves, and the final curves are highlighted in blue. Arrows show the direction of evolution in time. In (a) and **(c)**–(e), curves are shown at every 2𝚃 for a simulation time horizon of T=200𝚃. The parts of the curves which lie outside the species’ range (*n*_*i*_ < 0.02) are made transparent. The ranges converge to an equilibrium state, with a limited region of sympatry formed in the middle of the habitat. Graph (b) shows that the species evolve character displacement over the region of sympatry.

Interspecific competition and gene flow along an environmental gradient jointly contribute to the evolution of character displacement (difference between trait means) and range borders between two species. For competitively identical species, this was first shown by Case and Taper [20] under the constant trait variance assumption, and then confirmed by Shirani and Miller [24] for the case where trait variance is free to evolve. Since understanding the underlying mechanisms of character displacement and formation of range borders is important for better understanding the stabilization mechanisms we describe in next sections, here we briefly demonstrate the range evolution of two competitively identical (equal) species in a linear environment. We parameterize the two species with identical ecological and evolutionary parameter values, set equal to the default values given in Table 1. We initialize the two species’ populations at opposite sides of the habitat and compute their range evolution under different levels of the steepness of the environmental gradient. The results are shown in Fig 1 and Fig B in S1 File.

When the environmental gradient is sufficiently steep, we observe (Fig 1) that range limits are formed at the interface between the two species. The initially allopatric species adapt to new habitat locations and expand their range. They become sympatric over a small region when they first meet at the middle of the habitat. Since both species are relatively well-adapted to the environment when they meet, their individuals over the region of sympatry have similar phenotypes. This initiates strong interspecific competition between the individuals, which induces character displacement in the populations within the region of sympatry. Character displacement further implies departure of the trait mean of each species from the trait optimum. The resulting maladaptation, along with the effects of interspecific competition, substantially decreases the population density of the two species at their interface (Fig 1a). This decline in density further increases the asymmetry in the core-to-edge gene flow. As a result, the maladaptive effects of gene flow to species’ peripheral populations is intensified, due to the term $2\;D_{i}\partial_{x}(\log n_{i})\partial_{x}q_{i}$ in Eq. (9), moving the species’ trait mean further away from the optimum trait within the region of sympatry.

As the spatial overlap between the two species increases, the reinforcing feedback between character displacement and asymmetric gene flow continues to induce further maladaptation and decline in density. Eventually, the amount of maladapted phenotypes in each species’ peripheral population becomes so large that it prevents further local adaptation and range expansion. As a result, a range limit is established for each species and a limited region of sympatry is formed at the middle of the habitat. The highlighted curves in Fig 1a and 1b show the steady-state population densities and trait means, respectively. Similar to the single-species simulation in Fig A1 in S1 Appendix, we also show the effects of dispersal and local effects of selection and competition on rate of change of trait mean (for the first species) in Fig 1c–1e. In comparison with the single-species case (with the same gradient), we see that the local adaptation rate by natural selection remains almost the same (Fig 1d vs Fig A1e in S1 Appendix), whereas the maladaptive effects of asymmetric gene flow is substantially intensified (Fig 1c vs Fig A1d in S1 Appendix).

Additional details of the range evolution of the species, including the spatial evolution of trait variances, can be found in a similar simulation shown in [24, Fig 7]. We further note that, although not shown here, intraspecific trait variance declines within each population over the region of sympatry. This reduced variation in phenotypes allows for strong competition, character displacement, and maladaptation; as required for the establishment of the range limits described above.

When the environmental gradient is not sufficiently steep, the maladaptive effects of asymmetric gene flow cannot reinforce the effects of character displacement to the level required to stop range expansion within a finite habitat. As Fig B2 in S1 File shows, in this case the overlap between the species continues to expand and the species eventually become sympatric over the entire habitat. The evolution of this complete sympatry is, however, much slower than the evolution of the limited sympatry as in Fig 1a.

It is worth noting that the evolution of character displacement requires the diversifying (disruptive) selection generated by competitive interactions between phenotypes to be stronger than stabilizing selection [20]. The character displacement shown in Fig 1b and Fig B in S1 File becomes more pronounced as the strength of stabilizing selection decreases [20, Fig 5]. This is because greater character displacement reduces competition, providing a fitness advantage. On the other hand, greater character displacement implies greater deviation from trait optimum and hence larger fitness load imposed by selection. When selection is weak, the balance between these opposing forces on fitness is reached at greater extents of character displacement. A similar effect is observed if competition is intensified by increasing V_1_ and V_2_. The simulations performed in [24] for faster growing species ($R_{1}=R_{2}=2\;𝚃$) also show a greater extent of character displacement. Here, we perform our simulations with (relatively) slowly growing species to follow our conceptualization of the range evolution of montane birds.

### 3.3. Range limits in linear environments: Stable or unstable?

Our results show that in a linear environment (see Box 1), the range limits formed for competitively identical (equal) species in Fig 1a are evolutionarily stable in a weak sense (see Box 2). If, for example, we initialize the downslope species at the same location as in Fig 1a, but displace the initial population of the upslope species upward, then the distribution of the two species will converge to a different equilibrium, no matter how small the displacement in the initial population of the upslope species is. This new equilibrium will look essentially the same as the equilibrium shown in Fig 1a, but with a region of sympatry and range limits that are shifted upslope (see graph (f) of Fig 8 in Box 2). Therefore, depending on where the populations are initially centered, their equilibrium range limits will settle at different locations. This further implies that the evolutionary range dynamics of the two species has a continuum of equilibria, shown schematically in graph (a) of Fig 8 in Box 2.

The weakly stable range limits formed in Fig 1 are not robust against environmental disturbances (Box 2) such as climate change that cause permanent changes in the trait optimum, no matter how small the disturbance amplitudes are. To see this, we initialize our simulation with the equilibrium populations of Fig 1a, and then gradually shift up the line of trait optimum by α=2.5𝚀 over a time course of $t_{r}=10\;𝚃$; see the schematics shown in graph (d) of Fig 8 in Box 2. We then keep the trait optimum unchanged for the rest of the simulation (T=500𝚃). We can conceptualize this environmental disturbance, for example, as a permanent change in the environment due to climate warming. The simulation results are shown in Fig C in S1 File. We observe that due to the climate-warming perturbation, the equilibrium distribution of Fig 1a is shifted upslope. Since $\partial_{x}Q=-0.5\;𝚀/𝚇$, the spatial shift in the range limits due to the 2.5𝚀 shift in the trait optimum appears to be exactly equal to 2.5/0.5=5𝚀. This direct proportionality further implies that the weakly stable range limits formed in Fig 1a will not be restored to their original location when a permanent change occurs in climate, no matter how small the change. However, it should be noted that the original range limits will be restored if the environmental disturbances occur as transient fluctuations (see Box 2) such that the trait optimum line is temporarily shifted up or down but eventually returns exactly to its original value. Unless large in amplitude, such transient disturbances that completely vanish in time, may not be of particular interest in studying stability of range limits.

**Box 2. Notions of stability.**

We use standard notions of stability. Although the dynamics of the evolutionary PDE model we use is understood in an infinite-dimensional function space, we avoid the technical language of indefinite-dimensional dynamical systems theory [41–43]. The equivalent notions from finite-dimensional dynamical systems [44–46] are sufficient for conceptual understanding of our results.

**Equilibrium:** An *equilibrium point*, or simply an *equilibrium* of a dynamical system is a steady state (fixed point) of the system: if the state of the system is initiated at an equilibrium, then it stays at the equilibrium for all time. For the PDE model that we use, a “point” is understood as a vector of functions (curves) in a space of all possible vectors of functions (e.g., curves of population density in graphs (e) and (f) of Fig 8 above). An equilibrium is *isolated* if it has a neighborhood in which there does not exist another equilibrium. Otherwise, the equilibrium is *non-isolated*.

**Perturbations and disturbances:** An equilibrium state of a system is established for a fixed (constant) set of system parameters and external forcing terms. Yet the parameters are often subject to *perturbations*, and the system receives external *disturbances*. An *additive* perturbation δR in a parameter R, for example, changes R to R+δR. We say a perturbation is *transient* if it vanishes in finite time or asymptotically as t→∞. Otherwise, we say the perturbation is *permanent*. The rectangular-shaped perturbation in graph (c) of Fig 8 above, for example, is a transient perturbation with amplitude α and ending (termination) time *t*_e_. The disturbance δQ in graph (d) of Fig 8, with rise time *t*_r_ and amplitude α, is a permanent disturbance.

**Attracting set:** A set of points 𝒜 is *attracting* if there exists a neighborhood 𝒩 of 𝒜 such that all trajectories (evolution of system states in time) starting from the points in 𝒩 eventually converge to 𝒜 as t→∞. The set 𝒜 (in purple) in graph (a) of Fig 8 gives an example.

**Stability:** An equilibrium is *(Lyapunov) stable* if all trajectories starting near the equilibrium stay close to it for all time. More precisely, an equilibrium $u^{*}$ is stable if for any neighborhood 𝒰 of $u^{*}$ there exits another neighborhood 𝒩 of $u^{*}$ such that all trajectories starting in 𝒩 remain in 𝒰 for all time. Note that a stable equilibrium is not necessarily attracting, and it may or may not be isolated. An equilibrium that is both stable and attracting is *asymptotically stable* (graph (b) of Fig 8). An asymptotically stable equilibrium is isolated. When an equilibrium is stable but is not attracting, we say it is *neutrally stable*. A particular case of neutrally stable equilibria is where a system has an attracting set 𝒜 composed of a continuum set of (infinitely many) equilibria, such as the set 𝒜 shown in graph (a) of Fig 8. Note that although 𝒜 is an attracting set, none of the equilibrium points in 𝒜 is attracting. Every neighborhood of an equilibrium $u_{0}^{*}$ in 𝒜, no matter how small it is, contains another equilibrium $u_{1}^{*}$. Trajectories starting form $u_{1}^{*}$ will not converge to $u_{0}^{*}$. To which equilibrium trajectories starting from the *domain of attraction* (attraction neighborhood) of 𝒜 will converge depends on the initial state (starting point) of the trajectory. Note also that, when the system shown in graph (a) of Fig 8 is at an equilibrium, a small perturbation (force) to the system causes the state of the system to transition to another equilibrium.

**Weakly stable range limits:** We say a species’ range, and correspondingly its range limit(s), is *evolutionarily stable in a weak sense*, or *weakly stable*, if the species’ population dynamics (and hence its population density) is at a neutrally stable equilibrium. The limited range of a solitary species formed in a linear environment due to the swamping effects of gene flow, as identified in [26], is an example of a weakly stable range. Graph (e) of Fig 8 shows, schematically, the equilibrium population densities $u^{*}$ corresponding to such a limited range formation. Depending on where the population is initially centered, the equilibrium population distribution will be centered at *c*_0_, *c*_1_, or any other locations on the geographic axis. Therefore, there exists a continuum of equilibrium population states with limited range, similar to the case shown in graph (a) of Fig 8. Transient perturbations of small amplitude will shift the species’ equilibrium range from one location to another location. Similarly, the equilibrium population density curves shown schematically in graph (f) of Fig 8, corresponding to the range limits formed between two competitively identical species, are weakly stable. The stable range limits shown by [20], or in Fig 1 of the present work, serve as examples.

**Strongly stable range limits:** If a species’ population dynamics is at an asymptotically stable equilibrium, we say the species’ range (or the range limits) are *evolutionary stable in a strong sense*, or *strongly stable*. Transient perturbations of sufficiently small amplitude, although temporarily move the species range limits, will not cause a permanent shift in the species’ equilibrium range. Once the perturbation vanishes, the species’ population will return back to the same equilibrium state it was initially at before the perturbation occurred. As we show, the stability of the range limits formed at environmental knees and wiggles is strong.

Although the weak stability of the range limits formed in Fig 1 makes them quite sensitive to disturbances, the main drawback of such range limits is that they are not generic. That is, in a (almost) linear environment, a coevolutionary equilibrium state such as the one shown in Fig 1a—with a fraction of each species’ range being sympatric and the remaining being allopatric—does not exist, almost surely. Our simulations show the existence of such equilibrium states only for the special case of “identical” (or equal) competitors, which is unlikely to ever be the case in the real world. When one of the species is competitively stronger than the other, it constantly pushes the interface between the two species towards the weaker species. This means that the competitively formed range limits will not be evolutionarily stable. In fact, since the environment changes linearly, every point in the habitat imposes the same level of selection pressure on both species. Therefore, the weaker species does not receive any advantage over the stronger species from abiotic environmental factors, which could possibly counterbalance the competitive difference between the species and stabilize the range limits. To demonstrate this instability of the range limits for unequal competitors via simulations, we initialize the populations with the equilibrium curves of Fig 1 and increase each of the parameters R_1_, D_1_, V_1_, and K_1_ of the downslope species by only one percent. The simulation results shown in Fig D in S1 File verify the instability of the range borders and their constant move towards the weaker species.

In the real world, the competitive difference between two species can be significant. As in [24], our simulations show two possible evolutionary outcomes of range evolution in a linear environment: complete exclusion of the weaker species, or marginal existence of the weaker species at the vicinity of the habitat boundary. When one of the species is much stronger than the other, it completely excludes the other species. This is shown in Fig E in S1 File, where the downslope species is dominant due to its significantly larger maximum growth rate; $R_{1}=1.2\;{𝚃}^{-1}$ versus $R_{2}=1\;{𝚃}^{-1}$. When the competitive difference is smaller, as in Fig F in S1 File, an evolutionarily stable state of marginal coexistence can eventually form as the interface between the two species approaches the habitat boundary. In this case, the weaker species survives at low density at the vicinity of the boundary. This is because when this species is eventually pushed against the boundary, it starts gaining advantage from its range contraction and density decline. Since there is no influx of phenotypes from the boundary, the contraction in range (core) results in less migration load [47] caused by gene flow on the peripheral population of this species (where it interfaces with the stronger species). Additionally, the decrease in density reduces the intraspecific competition load on this population. The stronger species, however, does not enjoy these advantages. As a result, the decrease in the fitness load on the weaker species compensates for its moderate competitive weakness and allows the species to survive.

Based on our discussion above, we conclude that when the environment changes almost linearly in space, the range limits formed by the interaction between interspecific competition and gene flow are unlikely to be evolutionarily stable. Below, we show how environmental knees and wiggles can strongly stabilize such range limits.

### 3.4. Environmental knees and wiggles can stabilize range limits

The key nonlinearity in the environment that effectively stabilizes competitively formed range limits is a sharp change in the steepness of the gradient in trait optimum. In its simplest form, such a nonlinearity appears as a single knee in the environment. In its possibly more pervasive form, the nonlinearity may appear as two adjacent knees, forming a wiggle (see Box 1). We should note that changes in the spatial profile of the trait optimum at environmental knees and wiggles do not necessarily associate with a change in the productivity of individuals. Therefore, the effects of knees and wiggles on species’ range dynamics described here are distinct from those of gradients in productivity.

**Fig 2:**
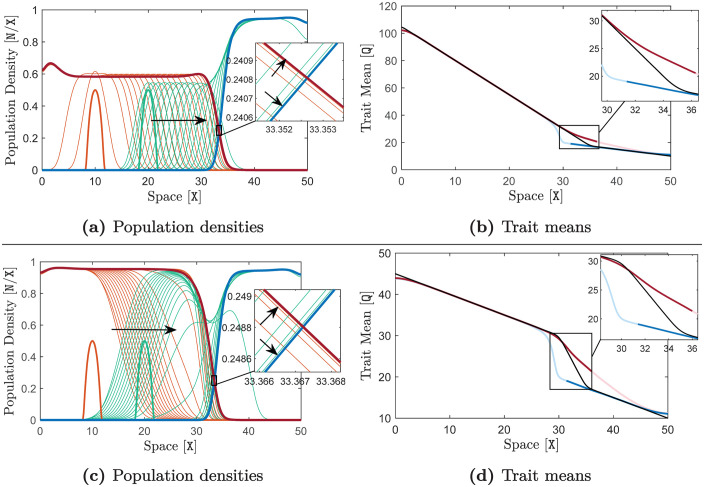
Formation of stable range limits at environmental knees and wiggles. Here, $R_{1}=1.1\;{𝚃}^{-1}$, $R_{2}=1\;{𝚃}^{-1}$, and the rest of the parameters take their typical values given in Table 1. The results shown in (a) and (b) in the upper panel are associated with a habitat with an environmental knee located at x=35𝚇, at which the slope of spatial variations in the trait optimum changes sharply from −2.5𝚀/𝚇 to −0.5𝚀/𝚇. The results shown in (c) and (d) in the lower panel are associated with a habitat with an environmental wiggle, at which the slope of trait optimum switches sharply from −0.5𝚀/𝚇 to −2.5𝚀/𝚇 between the two knees of the wiggle located at x=30𝚇 and x=35𝚇. The simulations are performed for a time horizon of T=1500𝚃 and curves of population density are shown in (a) and (c) at every 30𝚃. The same description as given in Fig 1 holds for the curve colors and arrows. The curves highlighted in red and blue are associated with the equilibrium state of the populations, reached (approximately) at the end of the simulations (t=1500𝚃). The highlighted equilibrium curves in (a) and (c) represent the evolutionarily stable range limits formed at the environmental knee and wiggle, respectively. The corresponding curves of equilibrium trait means are shown in (b) and **(d)**, where the black lines show the environmental trait optimum Q**.** The equilibrium curves of trait mean are made transparent over the regions where population densities are approximately zero.

We first show that environmental knees can stabilize competitively formed range limits, which are otherwise unstable in a linear environment. For this, we simulate an environmental trait optimum that is linear everywhere, except at a knee located at x=35𝚇. The steepness of the gradient changes sharply at the knee from 2.5𝚀/𝚇 to 0.5𝚀/𝚇; see the black line in Fig 2b. We let the downslope species be stronger, with $R_{1}=1.1\;{𝚃}^{-1}$ versus $R_{2}=1\;{𝚃}^{-1}$. We initialize both species on the same side of the habitat, downslope of the knee, and let their range evolve for an evolutionary time horizon of T=1500𝚃. The results are shown in the upper panel of Fig 2. We first note that in Fig 2a the species’ population density over the steep (downslope) part of the habitat is substantially lower than the environment’s carrying capacity. As we describe in Appendix D in S1 Appendix for a solitary population, this is because the phenotypic load imposed by natural selection is strong when the gradient is steep. However, the steepness 2.5𝚀/𝚇 we chose for this part of habitat is still sufficiently smaller than the critical (extinction) steepness $|\partial_{x}Q{|}_{\max}$ given in Appendix D in S1 Appendix. Based on the estimates provided in [24], an environmental gradient of 2.5𝚀/𝚇 is biologically plausible as well, though it is very steep.

Fig 2a shows that the species’ competitively formed range limits converge to an equilibrium state near the environmental knee. Initially, the upslope species expands its range in both directions; both downslope towards the stronger species and upslope towards the knee. When this species crosses the knee, it experiences a much shallower gradient (0.5𝚀/𝚇) and hence much weaker migration and genetic loads. As a result, it grows to high density over this part of the habitat and expands its range relatively quickly up to the habitat boundary. On the other side, when this species meets the downslope species, a region of sympatry is initially created between the two species. Since the downslope species is stronger, it pushes the interface between the species towards the knee. The interface is eventually stabilized at an equilibrium near the knee. The curves highlighted in red and blue in Fig 2a show the equilibrium (steady state) population distribution of the species.

The mechanism of range limits stabilization at an environmental knee relies on the contrast that the sharp change in the steepness of the gradient creates in the level of maladaptive gene flow to species’ peripheral populations. As the interface between the two species approaches the knee, the levels of asymmetric gene flow from central to peripheral populations of the two species change differently. The central population of the upslope species has already established itself in the region upslope of the knee, and hence is well-adapted to the shallow gradient there. As a result, the peripheral population of this species suffers less disruptive effects of gene flow as the interface approaches the knee. By contrast, the central population of the downslope species is adapted to the steep gradient downslope of the knee. Therefore, the phenotypes brought by dispersal to the peripheral population of the downslope species are significantly greater than the average local phenotype. As a result, the trait mean of the downslope species significantly deviates (upward) from the trait optimum near the knee, causing a substantial fitness load on the peripheral population of this species. The curves of trait means in Fig 2b show the resulting difference between the (steady-state) local adaptation of the peripheral populations of the two species. Since competition between the two species takes place within their overlapping peripheral populations, the advantage that the upslope species gains from reduced maladaptive effects of gene flow eventually compensates for its competitive weakness. This balances the competition between the two species and stabilizes their range limits as an equilibrium near the knee.

The coevolutionary mechanism of range stabilization we described above suggests that the competitively formed range limits at environmental knees are evolutionarily stable in a strong sense (see Box 2). The peripheral populations of the two species at their steady-state interface formed near a knee are subject to two opposing forces. If a transient perturbation slightly moves the interface upslope, then the fitness advantage that the upslope species has due to its local adaptation to the environment upslope of the knee gives this species the competitive strength needed to push the peripheral population of the downslope species (and hence the interface) back to the equilibrium location. If the interface is moved downslope by a perturbation, then the intrinsic competitive dominance of the downslope species works to push the interface back to the equilibrium location. Therefore, the presence of these two opposing forces ensures that the equilibrium state (point) of the species’ range evolution is isolated (see Box 2). Unlike the weakly stable range limits in Fig 1, the range dynamics shown in Fig 2a also implies that the equilibrium population distributions are not very sensitive to where the populations are initially centered. The simulations provided below for the similar case of range stabilization at environmental wiggles further demonstrate the strong stability of the range limits at environmental knees.

To demonstrate the range stabilization dynamics at environmental wiggles, we simulate an environmental trait optimum that is linear everywhere with slope −0.5𝚀/𝚇, except at a wiggle where its slope switches sharply to −2.5𝚀/𝚇; see the black line in Fig 2d. Same as the simulations we performed above for an environmental knee, we let the downslope species be stronger, with $R_{1}=1.1\;{𝚃}^{-1}$ versus $R_{2}=1\;{𝚃}^{-1}$. We initialize both species on the same side of the habitat, downslope of the wiggle, and compute their range evolution for T=1500𝚃. The results are shown in the lower panel of Fig 2. We first note that despite the sharp transition in the steepness of the environmental gradient at the wiggle, the upslope species can still adapt to and cross the wiggle; thereby expanding its range indefinitely up to the upslope habitat boundary. This ensures that the wiggle is not a physical barrier to range expansion.

Fig 2c shows that the spatial distribution of the species converges to an equilibrium, at which the range limits are stabilized near the upslope knee of the wiggle. When the interface formed between the species enters the wiggle’s transition zone, the substantially steeper environmental gradient of the wiggle intensifies the interactions between species’ population density and gene flow. These intensified interactions enhance character displacement within the wiggle, sharpen the interface between the species, and slow down the advancement of the interface. The interface is eventually stabilized near the upslope knee of the wiggle.

The mechanism through which environmental wiggles stabilize range limits is essentially the same as the stabilization mechanism at knees. In fact, only one of the knees of the wiggle (the upslope knee in Fig 2) plays the main role in stabilizing the limits. The steepness change at the other knee provides further support for the stability of the limits, depending on how close the two knees are to each other. In the results shown in Fig 2c, the downslope species can grow to high density in the moderate gradient below the downslope knee. Since the wiggle is relatively short, the disruptive effects of asymmetric gene flow from the high-density core of this species still remain in effect in its peripheral population. The increased migration load on this population, which competes with the other (intrinsically weaker) species near the upslope knee, increases the chance of range limit stabilization. The effects of the downslope knee on increasing the migration load on the peripheral population of the downslope species can also be verified by comparing the steady-state levels of character displacement in Fig 2b and 2d. The extent of character displacement at x=35𝚇 in Fig 2b (where the single environmental knee is located) is equal to 4.83𝚀, whereas as the character displacement at the same location in Fig 2d (where the upslope knee of the wiggle is located) is equal to 5.24𝚀.

Since range stabilization mechanisms at environmental knees and wiggles are essentially the same, our previous discussion on the stability of the range limits at environmental knees also suggests that the range limits formed at wiggles are evolutionarily stable in a strong sense (Box 2). Due to the opposing eco-evolutionary forces acting on the interface of the two species when it gets stabilized at the wiggle, the equilibrium state that the species’ range evolution converges to is isolated. Convergence to this equilibrium is fairly insensitive to the initial distribution of the two species. Fig G in S1 File shows, for example, that the population distributions converge to the same equilibrium as in the lower panel of Fig 2 even in the case where the two species are initialized at opposite sides of the wiggle. Moreover, simulations of transient perturbations (Box 2) in each of the characteristic parameters of the species confirm that the equilibrium state of Fig 2 is precisely restored after being temporarily disturbed by the perturbations. See, for instance, the results shown in Fig I in S1 File for additive perturbations of 20 percent in amplitude that last for 200𝚃 in each of the parameters R_1_, D_1_, V_1_, and K_1_ of the downslope species. In the results presented in the next section we also show that the stability of the strongly stable range limits formed at wiggles (as well as knees) is fairly robust against permanent climatic disturbances.

At this point in presenting the results, it is important to clarify the role that the inclusion of Allee effect in the model plays in maintaining the stability of the range limits. For this, we repeat the simulation shown in Fig G in S1 File, but in the absence of Allee effect. The results are shown in Fig H in S1 File. Without the Allee effect, the population growth rate (2) becomes purely logistic. As a result, the growth rate of a population becomes maximum when its density approaches zero. This implies that any infinitesimal population of a competitively stronger species that initially coexists with the weaker species, or gradually leaks to the region where the weaker species is established, can grow to significantly large densities and disturb the stability or even the existence of the range limits. The continuum mean-field framework of our model indeed allows for the spread of the populations in infinitesimally low densities. As shown in Fig H in S1 File, when simulation is performed for a sufficiently long time, the growth of the infinitesimal “tail” of the stronger species eventually results in destabilization and removal of the range limits. In the presence of the Allee effect, however, infinitesimal populations have negative growth rates. Even though they can exist and even spread in the mathematical sense, they cannot grow to large densities to disturb range limits.

**Fig 3:**
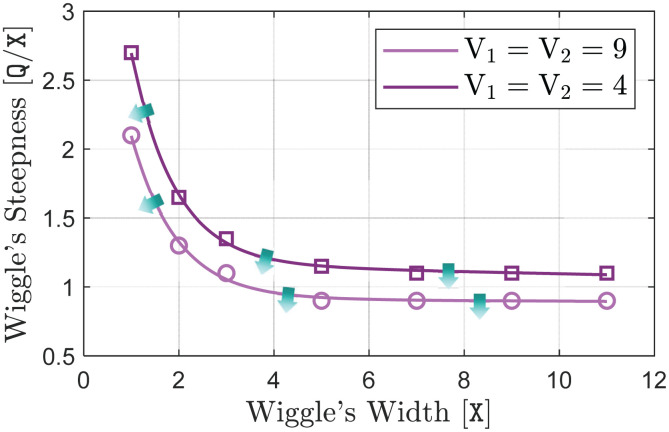
Critical wiggle parameters for stabilizing the range limits. To identify the critical wiggle parameters for stability of the range limits, the simulation associated with the lower panel of Fig 2 is repeated here for different values of steepness and width of the wiggle. The slope of the environmental gradient outside the wiggle is kept fixed at the same value −0.5𝚀/𝚇 as used in Fig 2. To find the critical wiggle parameters different values are chosen for the wiggle’s width within a reasonably wide range. A step-wise search (step-wise changes of step size 0.05𝚀/𝚇 in the steepness) is then performed at each width value to find the critical value of the wiggle’s steepness, below which the wiggle fails to stabilize the range limits. To do the searches, the simulation is run at each step (i.e., for each (width, steepness) pair) for a period of T=5000𝚃 and convergence to an equilibrium is checked at the end of the simulation. The critical parameter values obtained through the searches are marked in the graph. The critical values marked by circles are associated with simulations with the strong level of competition ($V_{1}=V_{2}=9\;{𝚀}^{2}$) used throughout the paper. The critical values marked by squares are associated with a more moderate level of competition ($V_{1}=V_{2}=4\;{𝚀}^{2}$). The curves connecting the points are computed by interpolation. Wiggles that are shallower or narrower than the critical values marked in the graph (lie below the curves) cannot stabilize the range limits (i.e., no convergence to an equilibrium is verified at the end of the simulation). Arrows indicate transition from stable (dark) to unstable (light) range limits as the wiggle parameters change across the curves.

We conclude this section by exploring the parameter values that make a wiggle effective enough to stabilize the range limits. In general, whether or not a wiggle will stabilize the range borders between two competing species depends on all characteristic parameters of the species that affect their relative competitive strength, the steepness and width of the wiggles, and the steepness of the environmental gradient outside the wiggle. These essentially include all parameters of the model. Since the comprehensive exploration of the entire multi-dimensional parameter space of the model is infeasible, we focus on the effects of the steepness and width of the wiggles. Specifically, we set the parameters of the species equal to those used in Fig 2 and fix the slope of the environment outside the wiggle at −0.5𝚀/𝚇. We then let the width and steepness of the wiggle change over a reasonably wide range of values, and identify the critical width and steepness values at which a transition (bifurcation) occurs in the wiggle’s effectiveness. We perform this analysis for two different levels of competition intensity, determined by the values of phenotype utilization variances. The results are shown in Fig 3.

For the given parameter values, wiggles shallower or narrower than the ones on the curves shown in Fig 3 cannot stabilize the range limits, whereas wiggles steeper or wider than those can. When competition between species is more intense (larger values of V_*i*_), the range limits can be stabilized by a shallower or a narrower wiggle. The results shown in Fig 3 suggest that narrow wiggles, with a width approximately less than 2𝚇, can stabilize range limits only if they are very steep. This is because the width of narrow wiggles is comparable to the mean dispersal distance of the individuals in one generation time. As a result, a significantly large portion of the peripheral population of the stronger species will cross a narrow wiggle by dispersal before being eliminated by natural selection. Unless this population receives a substantial level of maladaptive gene flow due to the wiggle being very steep, it will be able to outcompete the weaker species in the shallow environment adjacent to the wiggle and push the range limits out of the wiggle. Sufficiently wide wiggles, on the other hand, can stabilize range limits even if they are only moderately steep; for example, with a steepness as low as 0.9𝚀/𝚇, compared with the outside-wiggle steepness of 0.5𝚀/𝚇 used in the results of Fig 3. We see further that, when the wiggles are sufficiently wide, their minimum steepness required for range stabilization is almost independent of their width. This is because in wide wiggles only one of the knees (upslope knee here) plays the essential role in stabilization. However, as we discussed for the results shown in Fig 2, close adjacency of the knees can result in a more pronounced character displacement at the stabilizing knee of the wiggle. Greater character displacement then provides greater robustness for the stability of the range limits against environmental changes, as we discuss below. Based on these observations, the curves shown in Fig 3 suggest that a wiggle width approximately equal to 4𝚇 is almost optimal for the species’ competitive difference that we simulated: it is wide enough to allow for lower stabilizing steepness, and is narrow enough to provide better robustness against disturbances. The wiggle width 5𝚇 that we used in our other simulations is close to this optimal value.

### 3.5. Climate change and stability of the range limits

The strong stability of the range limits formed as a coevolutionary equilibrium at environmental knees and wiggles is robust against permanent climatic disturbances, provided they are sufficiently small in amplitude. Here, we only show the robustness at wiggles, noting that the robustness at knees hold similarly. Moreover, we only consider permanent climate-warming disturbances. Robustness against climate-cooling disturbances are similar, but with the “upslope” and “downslope” attributes interchanged. As in previous simulations, we present the results for a trait optimum that decreases as species move upslope. The same results hold if individuals are represented by a trait that increases with elevation, such as wing length in birds [48]. In our conceptualization of an elevation-dependent temperature gradient, the curves of trait optimum will have a positive slope in this case and climate warming will result in the curves to shift downward.

**Fig 4:**
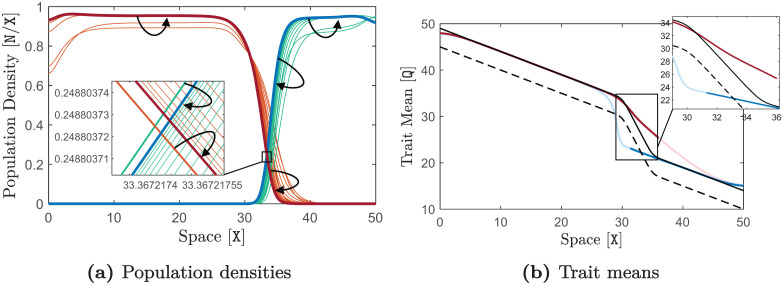
Robustness of the stability of the range limits against moderate climatic disturbances. The simulation is performed using the same model parameters as used in Fig 2. The equilibrium curves obtained at the end (t=1500𝚃) of the simulation shown in Fig 2 are used as the initial curves here. Starting from t=0𝚃, the curve of trait optimum Q is gradually shifted up by 4𝚀 over a time course of 10𝚃 and remains unchanged afterwards. That is, we add to Q a disturbance δQ of the form shown in graph (d) of Fig 8 in Box 2, with amplitude α=4𝚀 and rise time $t_{r}=10\;𝚃$. The initial curve of Q is shown by the dashed black line in **(b)**, and the completely shifted curve after t=10𝚃 is shown by the solid black line. The simulation is performed for a time horizon of T=500𝚃. Curves of population density are shown at every 6𝚃 in **(a)**, and the final curves obtained at t=500𝚃 are highlighted. The final curves of trait mean obtained at t=500𝚃 are shown in **(b)**. The same description as given in Fig 1 holds for the curve colors. We observe that the range limits remain stable under the climate-warming disturbance applied at the beginning of the simulation. That is, the limits converge back to the same initial equilibrium after the time course of the perturbation is complete.

To perform our analysis, we initialize our simulation with the equilibrium populations of Fig 2 whose range limits have been stabilized at the wiggle. We simulate a climate-warming disturbance by gradually shifting up the curve of trait optimum Q by an amplitude α=4𝚀 over a time course (rise time) of $t_{r}=10\;𝚃$, starting from the beginning of the simulation. When the course of climate warming is complete, we fix the curve of Q at its new profile until the end of the simulation. See graph (d) of Fig 8 in Box 2 for a schematic of this environmental disturbance, δQ. We allow the species to evolve for T=500𝚃. The results are shown in Fig 4.

The range dynamics shown in Fig 4 confirms that the stability of range limits is robust against the climate-warming disturbance. At the beginning of the simulation, both species suffer some population losses as they fail to immediately adapt to the quickly changing environment. The peripheral population of the downslope (stronger) species initially has trait mean values greater than the environmental optimum, particularly near the upslope knee of the wiggle where the range limits are stabilized. Therefore, this population suffers less from the incremental changes in the optimum phenotype, and in fact enjoys the changes at some locations. As a result, the range limits are slightly pushed upslope by this species when climate warming is taking place. However, once the course of climate warming is complete and both species successfully adapt to the new environment, the range limits return to their initial equilibrium state.

**Fig 5:**
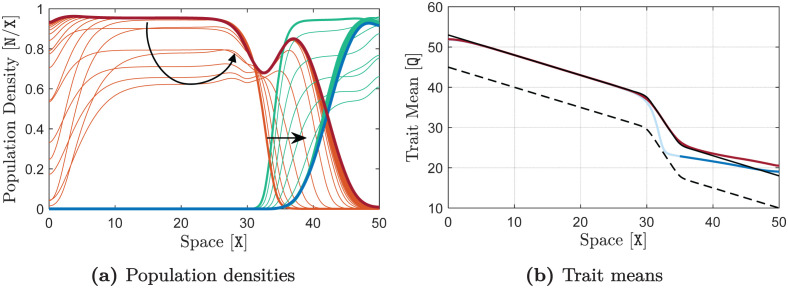
Destabilization of the range limits by a strong climate-warming disturbance when the downslope species is stronger. The same simulation as presented in Fig 4 is performed here, with the only difference being that the shift in the trait optimum takes a larger amplitude of α=8𝚀. The curves of population density are shown in **(a)** at every 2𝚃, up to the time t=100𝚃. The final curves are highlighted in red and blue at t=100𝚃, and their corresponding trait mean curves are shown in **(b)**. The same description as given in Fig 4 holds for curve colors and the dashed curve. Arrows indicate the direction of evolution in time. Due to the larger amplitude of the climate-warming disturbance, the initially stable range limits are destabilized, resulting in an upslope expansion in the stronger species’ range. Further continuation of the simulation up to T=2000𝚃 is shown in Fig J in S1 File, which shows that the weaker species will eventually be completely excluded from the habitat.

Climate warming can still destabilize the stable range limits in Fig 2 if it is sufficiently large in amplitude and occurs sufficiently fast. To see this, we repeat the same simulation as described above (Fig 4) but with a stronger disturbance amplitude of 8𝚀. The evolution of the species’ range for 100𝚃 is shown in Fig 5a. Here, the advantage that the peripheral population of the downslope (stronger) species has—due to its partial pre-adaptation to larger phenotype values within the wiggle—is significant enough to allow this species push the range limits entirely out of the wiggle before the upslope species finds a chance to sufficiently adapt to the warming climate. As a result, the range limits become destabilized and the downslope species succeeds in crossing the wiggle and expanding its range.

In general, there are several possibilities for the future range evolution of the species (with the downslope species being stronger) when their range limits formed at a wiggle are destabilized by climate warming. If there is another wiggle upslope to the previous wiggle, then the range limits can be stabilized at the new wiggle. In this case, the strong climate-warming disturbance causes an upslope shift in range limits from one wiggle to another wiggle. In the absence of another wiggle, the stronger species may eventually exclude the weaker one entirely from the habitat, or the species’ distribution may converge to an evolutionarily stable state of marginal coexistence at the vicinity of the habitat boundary; such as the state shown in Fig F in S1 File. Our further continuation of the simulation of Fig 5 up to T=2000𝚃 shows complete exclusion of the weaker species; see Fig J in S1 File. Another possibility, not shown here, is that the species’ ranges converge to an evolutionarily stable equilibrium at which the stronger species occupies the entire available habitat but the weaker species still survives at the vicinity of the wiggle with a low density and limited range.

**Fig 6:**
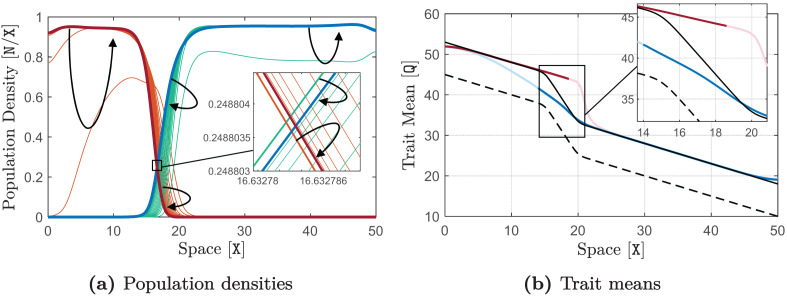
Robustness of the stability of the range limits against strong climate-warming disturbances when the upslope species is stronger. Here, $R_{1}=1\;{𝚃}^{-1}$ and $R_{2}=1.1\;{𝚃}^{-1}$, that is, in contrast with the results shown in Fig 2, the upslope (green) species is made stronger here. The rest of the parameters take their typical values given in Table 1. The environmental wiggle is located over the interval [15𝚇,20𝚇]. The formation of the stable range limits near the downslope knee of the wiggle is shown in Fig K in S1 File. Similar to the simulation associated with Fig 5, the equilibrium curves obtained at the end of the simulation shown in Fig K in S1 File are used as the initial curves here. The climate-warming disturbance δQ is also applied in the same way as it was applied in Fig 5, with the same amplitude of α=8𝚀 and rise time $t_{r}=10\;𝚃$. The simulation is performed for a time horizon of T=500𝚃 and curves of population density are shown in (a) at every 12𝚃. The final curves obtained at t=500𝚃 are highlighted in red and blue, and their corresponding trait mean curves are shown in **(b)**. The same description as given in Fig 5 holds for the curve colors, arrows and the dashed curve. We observe that, unlike the case where the downslope species was stronger (Fig 5), here the range limits remain stable even though the climate-warming disturbance is large in amplitude.

Finally, we show that if the upslope species is stronger, the stability of the range limits formed at a wiggle is robust even against strong climate-warming disturbances. For this, we repeat the simulations of Figs 2 and 5, but this time with $R_{2}=1.1\;{𝚃}^{-1}$ versus $R_{1}=1\;{𝚃}^{-1}$. That is, we first initialize the two species upslope of a wiggle and let their ranges be stabilized at the wiggle. The results are shown in Fig K in S1 File. Then, we use the equilibrium populations of this simulation to initialize a second simulation, where we apply a strong climate warming disturbance δQ of amplitude α=8𝚀 and rise time $t_{r}=10\;𝚃$, the same as what we did in Fig 5. We let the species’ range evolve for T=500𝚃. The results are shown in Fig 6. Similar to what we saw in Fig 5a, during the course of climate warming the range limits are (slightly) pushed upslope by the downslope species whose peripheral population is partially pre-adapted to larger phenotypes. However, since this species is competitively weaker, it cannot push the range limits out of the wiggle. Even if it can, possibly in the case of a stronger warming disturbance, the stronger upslope species will eventually push the limits back again to their original equilibrium in the wiggle (once the course of climate warming is complete and both species become adapted to the new climate).

**Fig 7:**
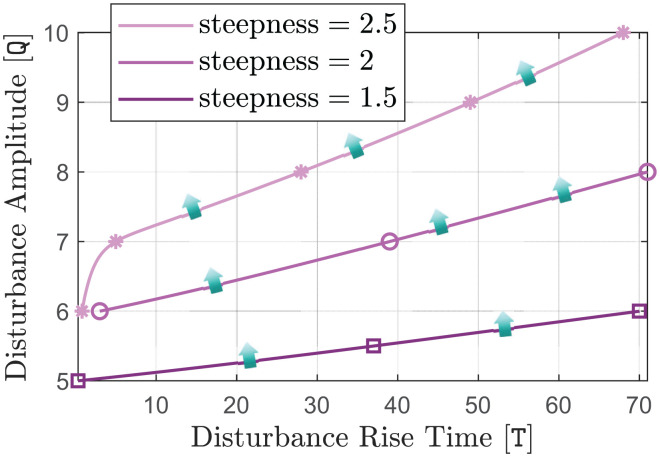
Critical climate-warming disturbances that can destabilize the range limits formed at environmental wiggles. The simulation associated with Fig 7 is repeated here for different values of the amplitude and rise time of the climate-warming disturbance, to identify the critical parameters that make the disturbance strong enough to destabilize the range limits formed at the wiggle. To ensure that the stability or instability of the equilibrium near the critical values is not affected by the habitat boundary condition, the habitat’s upslope boundary is extended to x=70𝚇, with the wiggle’s location remaining unchanged. To find the critical disturbance parameters, approximately, different values are chosen for the disturbance amplitude within a reasonably wide range of values. A step-wise search (step-wise changes of step size 1𝚃 in the rise time) is then performed at each amplitude value to find the critical value of the disturbance rise time, below which the disturbance can destabilize the range limits. To do the searches, the simulation is run at each step (i.e., for each (amplitude, rise time) pair) for a period of T=500𝚃 and divergence of the range limits from the initial equilibrium is checked at the end of the simulation. The critical parameter values obtained through the searches are marked in the graph. The critical values marked by asterisks are associated with simulations with the steep wiggle (steepness = 2.5𝚀/𝚇) used throughout the paper. The critical values marked by circles and squares are associated with a moderately steep and a shallow wiggle, respectively. The curves connecting the points are computed by interpolation. Disturbances that have higher amplitude or occur faster (have shorter rise time) than the critical values marked in the graph (lie above the curves) can destabilize the range limits. Arrows indicate transition from stable (dark) to unstable (light) range limits as the disturbance parameters change across the curves.

We conclude our results by exploring the disturbance parameter values α and *t*_r_ that make a climate-warming disturbance strong enough to destabilize the range limits formed at a wiggle; for the case where the downslope species is stronger. For this, we repeat the simulation used in Fig 4 for different values of disturbance amplitude (α) and rise time (*t*_r_). By checking the divergence (destabilization) of the range limits at the end of each simulation, we identify the critical values of these parameters: a disturbance that has higher amplitude or occurs faster than these critical values can destabilize the range limits. We perform our analysis for wiggles with three different levels of steepness but with the same width set equal to the almost optimal value 5𝚇. The results are shown in Fig 7.

**Fig 8:**
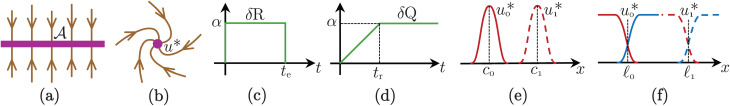
Schematic illustration of stability and parameter perturbation.

The curves of critical disturbance strength parameters in Fig 7 imply that steeper wiggles stabilize the range limits more strongly. That is, the stability of the range limits is more robust against permanent climatic disturbances when the wiggle is steeper. The steeper the wiggle is the stronger (more rapidly changing with higher amplitude) the disturbance must be to be able to destabilize the range limits. We further see, especially at shallow wiggles, that the destabilizing strength of the disturbance is more effectively determined by (is more sensitive to) its amplitude rather than its speed (rise time). In particular, no matter how fast the disturbance takes place, a minimum disturbance amplitude of about 5𝚀 is necessary to destabilize range limits; a value comparable to the maximum level of character displacement 5.24𝚀 that we computed for the coevolutionary equilibrium state in the lower panel of Fig 2.

## 4. Discussion

In this work we used a comprehensive deterministic model of species range evolution [24] to analyze the stability of range limits formed by interspecific competition. We proposed and studied environmental knees and wiggles as prevalent environmental nonlinearities that can robustly stabilize competitively formed range limits, which are otherwise unstable in (almost) linear environments; see Box 1 for the definition of linear environments, knees, and wiggles. We identified the contrast created in the level of maladaptive gene flow to species’ peripheral populations—when their competitively formed interface reaches an environmental knee—as the key factor in stabilizing the range limits. This contrast originates from the sharp change in the steepness of the environmental gradient occurring at a knee.

We note that Case and Taper [20] also discussed that the interface between two competing species will often become “attached” to places where environmental nonlinearities in the form of wiggles (or “kinks” as Case and Taper call them) exist. However, the mechanism they describe is only valid for the special and ecologically unlikely case where competitors are identical. This mechanism relies on the fact that the steep environmental gradient in a wiggle slows down the range expansion of the two species—initially located at opposite sides of the wiggle—thereby increasing the chance that they will meet within the wiggle. The range limits formed in this special case will be weakly stable (see Box 2). As also pointed out in [20], the equilibrium range limits formed at the wiggle through this “slowing-down” mechanism are sensitive to initial conditions. For example, if the two species are initialized at the same side of the wiggle as in our simulations, then the interface of the two (identical) species will simply stay at the location where the species first meet and will not move towards the wiggle. In contrast, the stabilization mechanism we identified in this work holds for the generic case of unequal species, results in range limits that are evolutionarily stable in a strong sense (see Box 2), and it essentially requires just a single knee in the environment (not necessarily a wiggle). This mechanism relies on the sharp change in the steepness of the gradient at a knee, rather than the reduction in species’ range expansion speed in the transition (steep) zone of a wiggle.

### 4.1. Implications for range limits and their stability

Our results have implications for the ongoing debate in ecology about how and when interspecific competition limits species’ ranges [49,50]. Our findings confirm that the interaction between interspecific competition and gene flow along an environmental gradient can set range limits [20,21,24]. However, we argue that when the effects of environmental conditions and biophysical constraints on the species (particularly those affecting a trait optimum) vary “almost” linearly in space, then range limits are generically unstable. An environment with knees and wiggles which are too mild to stabilize range limits (e.g., too shallow or too narrow wiggles; see Fig 3) is considered changing “almost” linearly. In such environments, and in the absence of physical barriers or additional interacting species, the interface between the species constantly moves towards the weaker species. This may eventually lead to complete exclusion of the weaker species, or to an evolutionarily stable state at which the weaker species marginally coexists with the dominant species at the vicinity of the habitat boundary; see Figs E and F in S1 File.

If the environment has a sufficiently steep wiggle ahead of the moving borders, or a knee at which the steepness of the environmental gradient changes significantly, then the borders eventually get stabilized at the wiggle or the knee. Environmental wiggles are likely more prevalent stabilizers of range limits, because species are less likely to colonize very steep gradients over a very wide habitat region near an isolated knee. Moreover, unlike solitary knees, wiggles serve as bidirectional stabilizers: species’ borders moving from downslope areas to a wiggle will get stabilized near the upslope knee of the wiggle, whereas the borders approaching from upslope areas will be stabilized near the downslope knee. If a wiggle is very narrow, of a width comparable to the mean dispersal distance of the species in one generation time, then it must be very steep to be able to stabilize the range limits; see Fig 3. The steepness of the wiggle must also be higher if the competitive difference between the species is greater. When the two species have more generalist individuals (larger values of V_*i*_), interspecific competition is intensified and range limits can be stabilized by a shallower or a narrower wiggle; see Fig 3.

We showed that the stability of the range limits formed at wiggles and knees is fairly robust against sufficiently small perturbations in the trait optimum, such as those caused by permanent climate change disturbances. The steeper the wiggle (or knee), the more robust the stability of the range limits; see Fig 7. Whether or not the range limits will be destabilized by strong climate changes depends on the type of the change (warming or cooling) as well as the relative position of the species across the wiggle. In our conceptualization of a trait that is positively correlated with an elevation-dependent temperature gradient (that is, smaller (cooler) trait values at higher elevations) sufficiently strong climate-warming disturbances destabilize the range limits when the downslope species is dominant. In this case, the strong disturbance allows the dominant species to cross the wiggle and make an upslope expansion in its range. By contrast, the stability of the range limits is robust, even against strong climate-warming disturbances, when the upslope species is dominant. The converse direction of effects hold for climate-cooling disturbances.

We should note that whether a wiggle with given width and steepness will stabilize the range limits depends on the level of competitive difference between the species as well as the steepness of the shallow gradient outside the wiggle. The competitive strength of the species relative to each other is determined by the combined effects of the differences between species in each and any of their characteristic parameters. Such differences lie in a high-dimensional parameter space that cannot be quantitatively characterized. Therefore, our general observations discussed above (as in Figs 7 and 3) should be interpreted qualitatively rather than quantitatively.

### 4.2. Infinitesimal populations and the stability of range limits

Our incorporation of Allee effect into the model was to prevent the growth and establishment of infinitesimal populations of the stronger species within the range of the weaker species, which would otherwise destabilize and remove the range limits. It is likely that the spread of such infinitesimal populations (Fig H in S1 File) in our simulations is an artifact of the continuum mean-field framework of our mathematical model. Extremely low values of population density are not biologically meaningful, because they would imply the existence of only a fraction of an individual in one unit of habitat volume. Moreover, mean-field representations often lose their validity for very low population densities. Despite these facts, we can still imagine that a small number of individuals of any natural populations will often find their way to leak to the territory of another competing population. Therefore, the formation of range limits by interspecific competition, and the stability of the limits, should in general require the presence of an ecological or evolutionary process that prevents the establishment of such small populations. Allee effect or random genetic drift could be examples of such processes. Also, small subpopulations that become detached from their core population often lack the sufficient genetic variation to adapt to new environments and are unlikely to survive environmental fluctuations.

### 4.3. Random dispersal and maladaptive core-to-edge gene flow

The mechanism of range limit stabilization at environmental knees and wiggles that we described in the present work relies on the maladaptive effects of asymmetric core-to-edge gene flow on adaptation of the peripheral populations; see Appendix D in S1 Appendix and Fig 1c. There is strong evidence that many species show declines in abundance towards their range edge; see for example [51,52] for North American birds. Evidence for asymmetric core-to-edge gene flow has also been found in nature [53–57]. The findings in [53], in particular, are consistent with the center (core)-periphery hypothesis [58], that genetic variation and demographic performance of a species decrease from the center (core) to the edge of its range. The spatial patterns of trait variance and trait optimum in our results (Fig A1 in S1 Appendix), as well as those shown in [24] are also consistent with this hypothesis.

Despite the available evidence, as discussed above, whether asymmetric gene flow consistently occurs from central to peripheral populations, and whether central-to-edge gene flow reduces mean fitness in edge populations, has been generally questioned [58–61]. This is in part because patterns of gene flow depend on multiple factors, such as population size, environmental context, genetic variation, and dispersal behavior [60]. The methodologies used to uncover patterns can also affect outcomes.

One potential source of difficulty, and possible misinterpretation, in examining the effects of gene flow is due to the contrasting effects of random gene flow: the swamping effects on population trait mean (Fig A1d in S1 Appendix) versus the enhancing effects on population adaptive potential (Fig A1e in S1 Appendix) [57,59,60,62]. Our analyses (Appendix D in S1 Appendix), as well as those in [24] and [28], show that the range expansion speed of a solitary species decreases as the steepness of the environmental gradient (hence the level of gene flow) increases. This implies that, in the model we used, the overall effects of random gene flow on range expansion capacity are not facilitative. Disentangling the contrasting effects of gene flow on range expansion speed is not straightforward. Yet, we propose that the reduction in expansion speed could be, in part, due to the fact that the trait variance evolves more slowly than the trait mean. Thus, the increased adaptive potential in steeper environments (due to the inflation in trait variance) cannot completely compensate for the swamping effects.

Other reasons for the scarcity of evidence for significant maladaptive gene flow could include that environmental gradients are often shallow, species exhibit reduced dispersal, or species disperse adaptively (non-randomly). When an environmental gradient is steep and dispersal is random, reduced dispersal can allow for faster range expansion by reducing maladaptive gene flow [28,63]. There is also empirical evidence confirming that many species in nature disperse adaptively to better habitat [64–68]. A phenotype-dependent dispersal strategy such as matching habitat choice [69,70] can indeed make gene flow adaptive [28,71–74]. However, such an adaptive dispersal strategy may evolve only when the environmental gradient is very steep [28].

When an environment has an isolated knee with a sufficiently wide region of steep gradient, the stable range limits formed at the knee may be destabilized if the stronger species evolves an adaptive dispersal strategy. Such a strategy releases the species from the competitive disadvantage of strong maladaptive gene flow to its interface with the other species. However, when range limits are stabilized at an environmental wiggle, evolution of dispersal strategies is less likely. Only the low-density peripheral population of the stronger species will occupy the steep gradient inside the wiggle, whereas the core of the species will reside in the shallow gradient outside the wiggle. Since the peripheral population constantly receives phenotypes from its core, which are adapted to the shallow gradient, it may not be able to evolve an effective dispersal strategy. This is a further reason why environmental wiggles might be more prevalent stabilizers of range limits, rather than isolated knees. In addition, it suggests that environmental wiggles could be the habitat locations where empirical studies examining maladaptive core-to-edge gene flow are most informative.

### 4.4. Environmental nonlinearities and range limits in nature

The importance of interspecific competition in limiting species’ ranges in nature is ultimately an empirical question, but our theoretical results shed light on this question. In particular, we highlight the crucial role of sharp changes (knees) or wiggles along environmental gradients in determining where interspecific competition can set stable range limits. Nonlinearities along environmental gradients, such as wiggles, would seem to be common in empirical systems. For example, ecotones between habitats are commonly observed. Some empirical observations also support an important role for step-like changes in environmental gradients to stabilizing species’ range limits. For example, closely related species often share a common range border at ecotones [75,76] and other transitional locations along environmental gradients [77]; consistent with the predictions of our analyses.

Our investigation of the environmental linearity and nonlinearity based on the optimum value that the environmental conditions impose on a trait allows for broader implications. The determination of the optimum trait is also subject to functional trade-offs and constraints [78,79]. For example, biomechanical, biochemical or physiological trade-offs may affect the trait optimum during the stages of development. Such trade-offs may vary nonlinearly even if the abiotic environmental conditions (such as temperature) vary linearly in space [80–86]. In particular, the nonlinearity in trade-offs may create a knee or wiggle in the trait optimum. Possible examples could be trade-offs between plants’ acquisitive and conservative strategies [87], drought tolerance and avoidance strategies [88], or drought resistance and recovery relationships [89,90], which can result in sharp nonlinear changes in functional traits along gradients in light or water availability. Within the framework of our study, an environment with such nonlinearities created by trade-offs is considered nonlinear, even if the environmental conditions appear to change linearly in space. Since the knees and wiggles caused by trade-offs impose the same nonlinearities in the selective gradient as we investigated throughout our work, the stabilization mechanism we described also hold for such nonlinearities.

The nonlinearities caused by functional trade-offs may respond differently to climatic changes in the environment. Assuming a temperature-correlated trait, in our simulations we modeled a climate-warming disturbance by shifting the whole profile of the trait optimum upward. The location of the wiggles remains unchanged under such a disturbance. When wiggles in the trait optimum are due to nonlinearities in trade-offs, a climate-warming disturbance could also move the location of the wiggle. If an environmental disturbance moves the wiggle towards the weaker species, then the range limits will shift and get stabilized at the new location of the wiggle, no matter how large the disturbance is; see Fig L in S1 File. If the disturbance causes the wiggle to move towards the stronger species, then the range limits will still get stabilized at the new wiggle location, provided the disturbance is sufficiently small; see Fig M in S1 File. However, if the disturbance is so strong that the wiggle moves rapidly and entirely inside the core of the stronger species’ population, then the range limits will get destabilized and the stronger species will push the weaker species to the habitat boundary. This is because in this case the disturbance effectively passes the stronger species across the wiggle.

### 4.5. Species’ response to climate change

Our results provide a perspective on how climate change will lead to changes in species’ ranges [91]. Most species are currently shifting poleward and upslope toward cooler environments, but many species have stable ranges or are even shifting towards warmer conditions [92,93]. One possible explanation for these divergent responses to climate change is that species’ interactions shape their ranges along environmental gradients. For example, interspecific competition between closely related species of tropical birds is an important factor limiting their elevational ranges [17,94,95]. The simple expectation is that species’ ranges will shift upslope as environments get warmer. However, our results suggest that this would hold only if the downslope (lower-elevation) species is the dominant competitor. Empirical evidence demonstrates this is not always the case [96]. Therefore, our results emphasize the importance of considering the competitive dominance between species when predicting changes in their range limits. When the environmental trait optimum varies almost linearly, a stronger competitor can expand its range regardless of whether it is located downslope to the other species (leading to an upslope shift in ranges) or upslope to it (leading to a downslope shift in ranges). If the range limits of the species are already stabilized at an environmental wiggle, then climate warming (if strong enough) can only result in an upslope shift in ranges, and that occurs only when the downslope species is dominant; see Figs 5 and 6. Conversely, climate cooling can only result in a downslope shift in ranges, and that occurs only when the upslope species is dominant. These patterns of shifts in ranges are independent of how species’ trait is correlated with temperature, whether it is positively correlated or negatively correlated. These findings particularly support the importance of measuring competitive ability of species empirically [51,97,98] in order to predict species’ responses to climate change.

### 4.6. Future research directions

The model we used does not include the effects of stochastic evolutionary processes such as genetic drift, which are thought to play an important role in setting range limits under certain conditions [62,99]. The mean fitness of small and possibly isolated populations at range margins can be substantially impaired by the effects of random genetic drift, that is, by increased probability of the fixation of deleterious alleles [62,100–103]. Genetic drift can also erode genetic variation in small populations, resulting in a reduction in their adaptive potential [104]. When competitively formed range limits are stabilized at an environmental wiggle (or a knee), the peripheral population of the stronger species may suffer a strong reduction in density along the steep gradient within the wiggle; see Fig 2. This makes the stronger species more sensitive to genetic drift at the environmental knee where it interfaces with the weaker species. Thus, genetic drift may strengthen the stability of the range limits by weakening the stronger species. An extension of our model that includes genetic drift could help test this hypothesis.

In our analyses, we assumed that the intrinsic competitive ability of the species (determined by their characteristic parameters, e.g., R_*i*_, V_*i*_, or D_*i*_) does not evolve in time. However, it is likely that in the real world species may evolve their competitive ability [97], which can result in different population outcomes [105–107]. If the evolution of competitive abilities is in the direction of releasing the species from interspecific competition, then no range limits may form by competition and the species may coexist all over the habitat. If competitive abilities are evolved to equalize the relative competitive strength of the species, without releasing them from competition, then the competitively formed range limits will eventually become (semi-) stable in the weak sense; noting that perfect equalization is unlikely to evolve. If the interface between the species reaches a sufficiently steep wiggle as they evolve to equalize their abilities, then their range limits gets strongly stabilized in the wiggle. The steep gradient within the wiggle substantially shortens the overlap between the species (sharpens the range borders) and reduces the peripheral population density of the stronger species; see Fig 2. This may significantly slow down or halt further evolution of the competitive ability of the species, since such abilities mainly evolve through overlapping populations. Therefore, we hypothesize that environmental wiggles not only can stabilize competitively formed range limits, but also they can halt evolution of species’ competitive ability by effectively isolating them from each other. The model we have used can be extended to include the evolution of competitive abilities in order to test our hypothesis.

In addition to the possibility of directly changing the optimum value of the focal trait, as we discussed above, ecological trade-offs often create negative genetic correlations between the associated traits via antagonistic pleiotropy or correlational selection [108–111]. Adaptation at range margins can be constrained by such genetic correlations when they become antagonistic to the direction of selection gradient. The effectiveness of these constraints can be evaluated by quantifying the genetic or phenotypic variance-covariance matrices [112]. As the environmental conditions change along a gradient, there may be a point in space at which spices’ adaptive potential changes sharply due to a trade-off. This creates a contrast in the adaptive potential of the species residing at opposite sides of the *genetically created knee*. In principle, the evolutionary effects of such a contrast are different from the effects of the contrast created in the maladaptive effects of gene flow across the environmental knees that we studied. Yet, the interactions between such effects and interspecific competition may still result in range limits that are “caught” at the genetically created knee [77,112]. However, given the fact that genetic variance-covariance matrices evolve in time [113] and the effects of the contrast in adaptive potentials may fade as species evolve over long evolutionary time, the evolutionary stability (Box 2) of such range limits is hard to predict intuitively. A multi-trait extension of our model could be developed to investigate this possible mechanism of range limit stabilization by trade-offs. The preliminary models of multi-trait adaptation and range evolution for solitary species [114,115] can help motivate the efforts.

## Supporting information

S1 File Supplementary figures. This file includes all supplementary figures referenced in the main text.
(PDF)

S1 Appendix This file includes all appendices referenced in the main text.
(PDF)
