# Supplementary material for: Environmental “knees” and “wiggles” as strong stabilizers of species’ range limits set by interspecific competition
Source: PLoS Comput Biol. 2026 Jun 15;22(6):e1014336. doi: 10.1371/journal.pcbi.1014336 (PMC13278590; doi:10.1371/journal.pcbi.1014336)
Supplement: S1 File — This file includes all supplementary figures referenced in the main text. (PDF) [file pcbi.1014336.s001.pdf]

Environmental “Knees” and “Wiggles” as Strong Stabilizers of Species’ Range Limits Set by Interspecific Competition (Supplementary Information)

Farshad Shirani[*†‡]   Benjamin G. Freeman[§]

List of Figures

A Intrinsic growth rate of a fully adapted population with Allee Effect 2
B Evolution of character displacement and region of sympatric coexistence for two competitively identical species in a shallow environmental gradient 3
C Permanent shift in the equilibrium region of sympatry and range limits in response to a permanent climate-warming disturbance 4
D Instability of the range limits for competitively unequal species in a linear environment 5
E Competitive exclusion 6
F Marginal coexistence 7
G Formation of stable range limits at an environmental wiggle when the two species are initialized at opposite sides of the wiggle 8
H Disruption of range limits stability in the absence of Allee effects 9
I Robustness of the stability of the range limits formed at wiggles against transient perturbations in species’ parameters 10
J Destabilization of the range limits by a strong climate-warming disturbance when the downslope species is stronger, leading to the exclusion of the upslope species . . 11
K Formation of stable range limits at an environmental wiggle when the upslope species is stronger 12
L Range shifts resulting from an environmental disturbance that moves the wiggle towards the weaker species 13
M Range shifts resulting from an environmental disturbance that moves the wiggle towards the stronger species 14

[*]School of Mathematics, Georgia Institute of Technology, Atlanta, GA 30332, USA
[†]Department of Physics, Emory University, Atlanta, GA 30322, USA
[‡]Department of Mathematics and Statistics, Georgetown University, Washington, DC 20057, USA
[§]School of Biological Sciences, Georgia Institute of Technology, Atlanta, GA 30332, USA

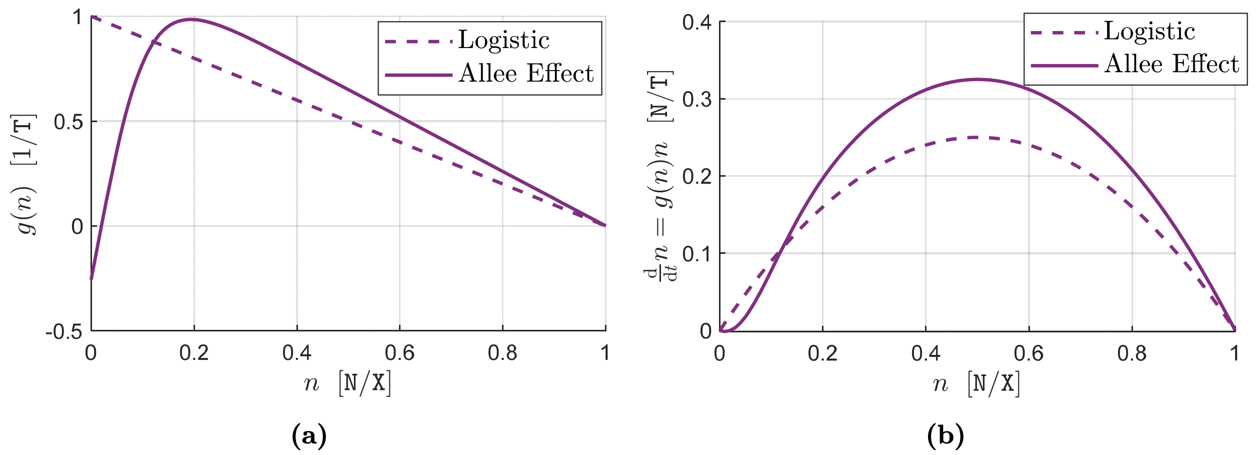

Fig A: Intrinsic growth rate of a fully adapted population with Allee Effect. For a solitary ($\mathrm{N} = 1$, hence we drop the numeration index i) population of individuals with phenotype p, which are completely generalist ($\mathrm{V} \to \infty$, which gives $\alpha(p, p') \to 1$ for all p and p'), and are perfectly adapted to the environment ($p = \mathrm{Q}$), the intrinsic growth rate given by equation (2) of the main text will depend only on the species' population density, as $g(n) = \mathrm{R}\,(1 - n/\mathrm{K})\,B(n)$. In the absence of dispersal (a local isolated population) and mutations, the rate of change in the density of this population will be $\frac{\mathrm{d}}{\mathrm{d}t}n = g(n)n$. The graphs of $g(n)$ and $\frac{\mathrm{d}}{\mathrm{d}t}n$ are shown in (a) and (b), respectively, both for the case where the growth rate is logistic, $B(n) = 1$, and the case where the population exhibits Allee Effect, $B(n) = \mathrm{B}_{\mathrm{max}}\left(\frac{1}{1+\exp(-(n-\mathrm{J})/\sigma)} - \frac{1}{2}\right)$. The parameter values used for computing the graphs are $\mathrm{R} = 1$, $\mathrm{K} = 1$, $J = 0.02$, $\mathrm{B}_{\mathrm{max}} = 2.6$, and $\sigma = 0.05$. Note that, as the curve with Allee Effect in (a) shows, for population densities below the critical density ($n < \mathrm{J}$), the intrinsic growth rate g is negative.

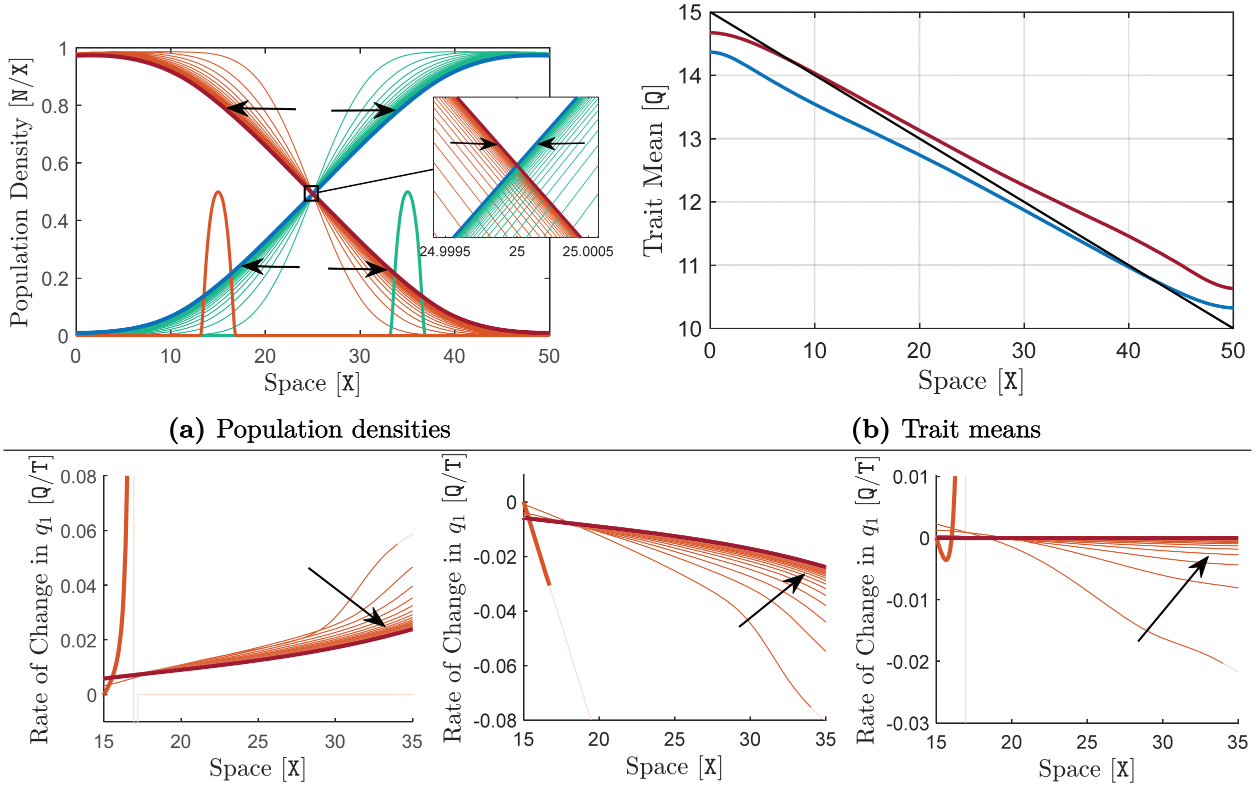

(a) Population densities

(b) Trait means

(c) Dispersal effects

(d) Local effects

(e) Total effects

Fig B: Evolution of character displacement and region of sympatric coexistence for two competitively identical species in a shallow environmental gradient. The same simulation as in Fig 2 of the main text is performed here, with the only difference being that the environmental gradient is set to be shallow. Specifically, the trait optimum Q is linear and decreasing, shown by the black line in (b), with a shallow gradient (slope) of $\partial_x \mathrm{Q} = -0.1$ Q/X. The same descriptions as given in Fig 2 of the main text hold for the quantities shown in the graphs, curve colors, and arrows. Here, curves in (a) and (c)–(e) are shown at every 20 T, for a simulation time horizon of $T = 1500$ T. The initially formed region of sympatry in the middle of the habitat continues to expand, rather slowly, until the species become completely sympatric over the entire habitat.

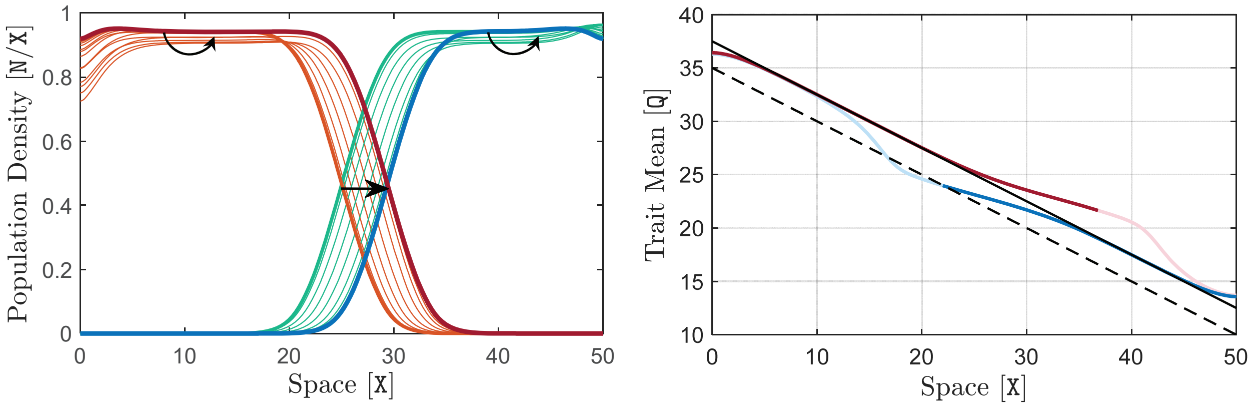

(a) Population densities

(b) Trait means

Fig C: Permanent shift in the equilibrium region of sympatry and range limits in response to a permanent climate-warming disturbance. The two species are competitively identical, with the same parameters as used in Fig 2 in the main text. The species are initialized at $t = 0$ T by the final solution curves (equilibrium state) obtained at the end of the simulation in Fig 2 of the main text (with $\partial_x \mathrm{Q} = -0.5$ Q/X). Starting from $t = 0$ T, the curve of trait optimum Q is gradually (linearly) shifted up by 2.5 Q over a time course of 10 T, and remains unchanged afterwards. That is, a climate-warming disturbance $\delta\mathrm{Q}$ is added to the initial curve of Q, where $\delta\mathrm{Q} = \alpha t / t_\mathrm{r}$ for $0 \leq t \leq t_\mathrm{r}$ and $\delta\mathrm{Q} = 0$ for $t > t_\mathrm{r}$, with disturbance amplitude $\alpha = 2.5$ Q and disturbance rise time $t_\mathrm{r} = 10$ T; see graph (*iv*) in Fig 8 in Box 2 of the main text. The dashed black line in (b) shows the initial curve of Q, and the solid black line shows the completely shifted curve after $t = 10$ T. The simulation is performed for a time horizon of $T = 500$ T. The same description as given in Fig 2 of the main text holds for the curve colors and arrows. Curves of population density are shown at every 2 T in (a) and the final equilibrium curves obtained at $t = 500$ T are highlighted in red and blue. The final curves of trait mean obtained at $t = 500$ T are shown in (b). We observe that the range limits converge to a new equilibrium state after the climate-warming disturbance takes place, resulting in a permanent upslope shift in range limits and the region of sympatry.

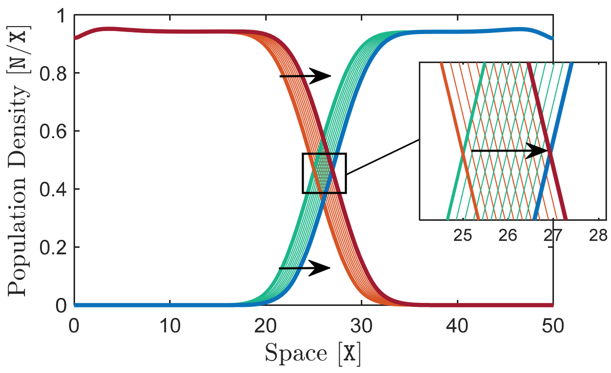

(a) Perturbation in R_1

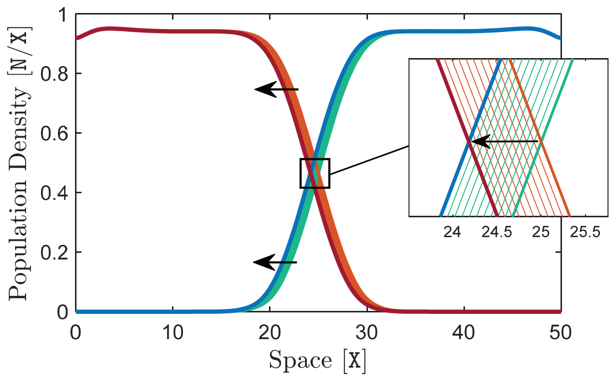

(b) Perturbation in D_1

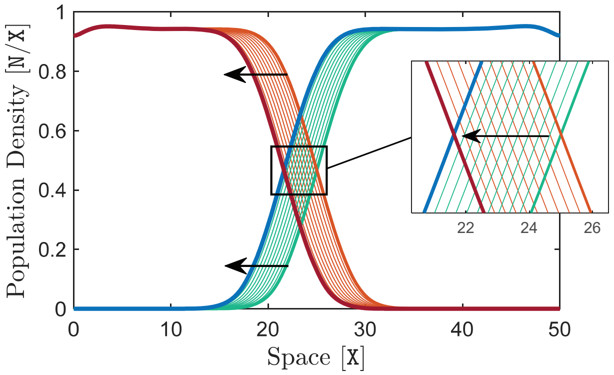

(c) Perturbation in V_1

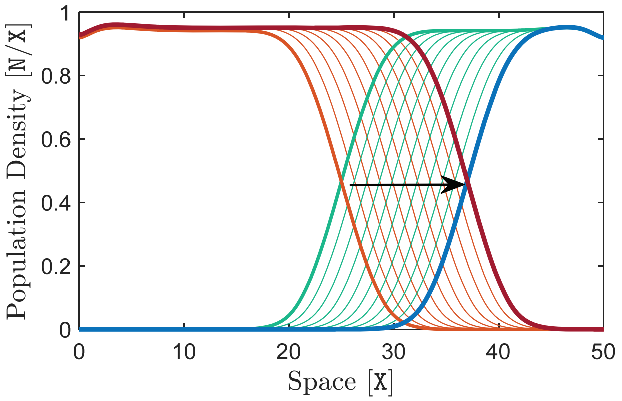

(d) Perturbation in K_1

Fig D: Instability of the range limits for competitively unequal species in a linear environment. The two species are initialized at $t = 0$ T by the final solution curves (equilibrium state) obtained at the end of the simulation in Fig 2 of the main text (with $\partial_x \mathrm{Q} = -0.5$ Q/X). Both species have the same parameter values, equal to those used in Fig 2, except for one of the parameters that is increased by only one percent for the downslope species in each panel. In (a), R_1 is increased to $\mathrm{R}_1 = 1.01$ T at the beginning of the simulation and is kept constant at this value for the entire simulation. This makes the downslope species slightly stronger than the upslope species. Similarly, in (b), D_1 is increased by one percent to $\mathrm{D}_1 = 1.01$ $\mathtt{X}^2/\mathtt{T}$, making the downslope species slightly weaker. In (c), V_1 is increased to $\mathrm{V}_1 = 9.09$ $\mathtt{Q}^2$, which makes the downslope species weaker. In (d), K_1 is increased to $\mathrm{K}_1 = 1.01$ N/X, making the downslope species stronger. In each case, the simulation is performed for a time horizon of $T = 1000$ T. Curves of population density are shown at every 100 T in each graph and the final curves at $t = 1000$ T are highlighted. The same description as given in Fig 2 of the main text holds for the curve colors and arrows. In all cases, we observe that a slight change in the competitive equivalence of the two species destabilizes (eliminates) the initial equilibrium (formed for the non-generic case of identical species), causing a constant shift in the range limits towards the weaker species.

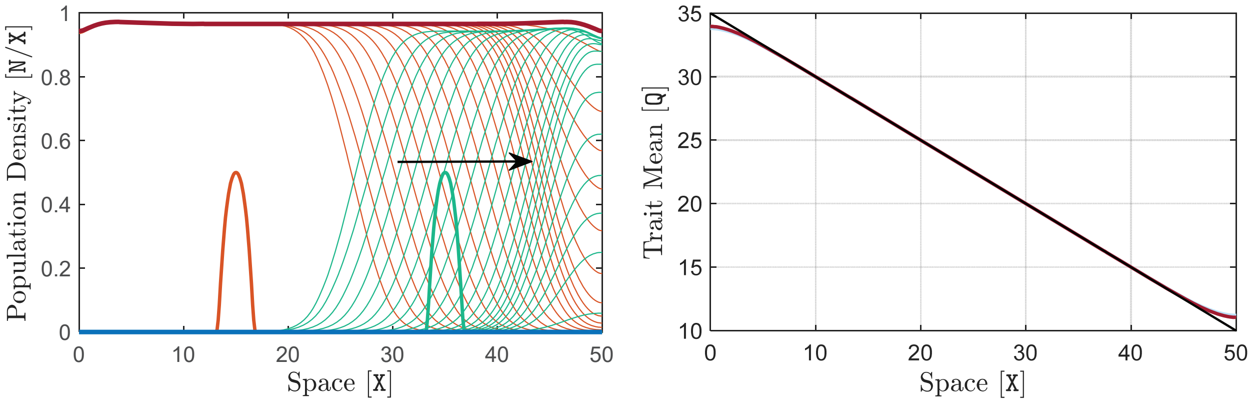

(a) Population densities

(b) Trait means

Fig E: Competitive exclusion. The downslope species is significantly stronger, with a maximum growth rate of $\mathrm{R}_1 = 1.2\ \mathtt{T}^{-1}$ versus the growth rate $\mathrm{R}_2 = 1\ \mathtt{T}^{-1}$ of the upslope species. The rest of the model parameters for both species are the same and equal to the values given in Table 1 of the main text. The curves of population density of the two species are shown in (a) as their range evolves in time. Arrows show the direction of evolution in time after the region of sympatry is formed between the species and starts moving upslope. The same description as given in Fig B holds for curve colors, and the highlighted curves in red and blue are the final curves obtained at the end of simulations. In (a), the curves are shown at every 40 $\mathtt{T}$ for a simulation time horizon of $T = 1500\,\mathtt{T}$. The downslope species eventually excludes the upslope species from the habitat. The curves of steady-state trait means obtained at the end of the simulation are shown in (b).

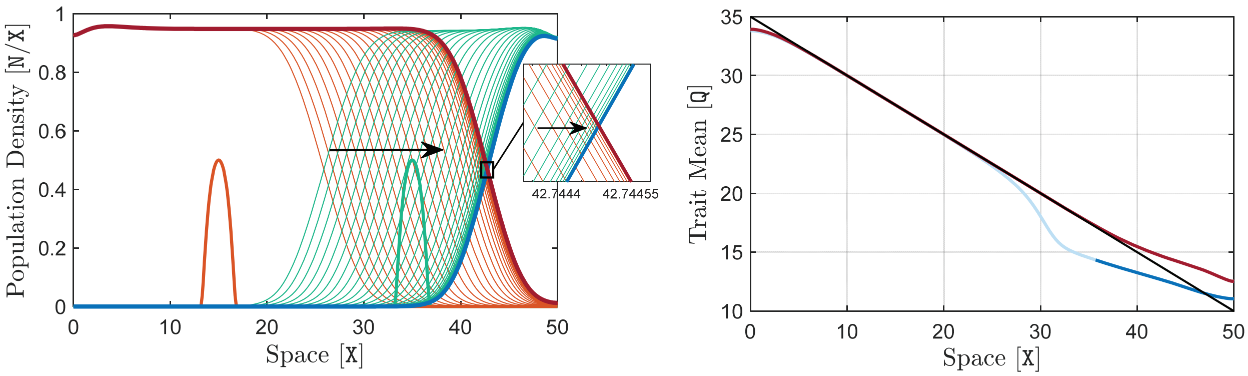

(a) Population densities

(b) Trait means

Fig F: Marginal coexistence. The downslope species is slightly stronger, with a maximum growth rate of $R_1 = 1.05\ \mathtt{T}^{-1}$ versus the growth rate $R_2 = 1\ \mathtt{T}^{-1}$ of the upslope species. The rest of the model parameters for both species are the same and equal to the values given in Table 1 of the main text. The curves of population density of the two species are shown in (a) as their range evolves in time. Arrows show the direction of evolution in time after the region of sympatry is formed between the species and starts moving upslope. The same description as given in Fig B holds for curve colors, and the highlighted curves in red and blue are the final curves obtained at the end of simulations. In (a), the curves are shown at every $100\ \mathtt{T}$ for a simulation time horizon of $T = 6000\,\mathtt{T}$. The species' population distribution converges to an evolutionarily stable equilibrium state, at which the upslope species survives marginally at the vicinity of the habitat boundary. The curves of steady-state trait means obtained at the end of the simulation are shown in (b).

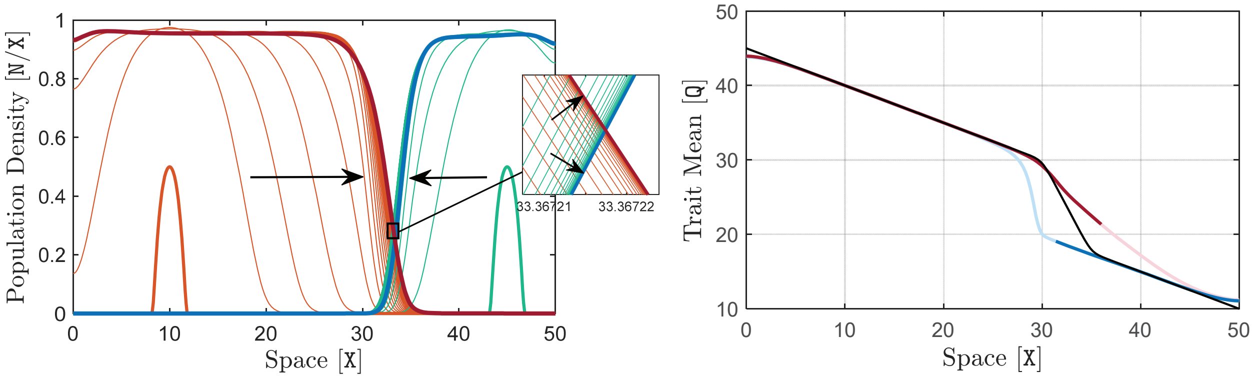

(a) Population densities

(b) Trait means

Fig G: Formation of stable range limits at an environmental wiggle when the two species are initialized at opposite sides of the wiggle. A similar simulation to that of the lower panel of Fig 3 in the main text is performed here, with the main difference being that the populations are initialized at the opposite sides of the wiggle. That is, $\mathrm{R}_1 = 1.1\ \mathtt{T}^{-1}$, $\mathrm{R}_2 = 1\ \mathtt{T}^{-1}$, and the rest of the parameters take their typical values given in Table 1 of the main text. The habitat has an environmental wiggle. The slope of the trait optimum switches sharply from $-0.5\ \mathtt{Q/X}$ to $-2.5\ \mathtt{Q/X}$ between the two knees of the wiggle located at $x = 30\ \mathtt{X}$ and $x = 35\ \mathtt{X}$. The simulation is performed for a time horizon of $T = 1500\ \mathtt{T}$ and curves of population density are shown in (a) at every $8\ \mathtt{T}$. The same description as given in Fig 2 of the main text holds for the curve colors and arrows. The curves highlighted in red and blue in (a) are associated with the equilibrium state of the populations, reached (approximately) at the end of the simulations ($t = 1500\ \mathtt{T}$). These curves represent the evolutionarily stable range limits formed at the environmental wiggle. The corresponding curves of equilibrium trait mean are shown in (b), where the black line shows the environmental trait optimum Q. The equilibrium curves of trait mean are made transparent over the regions where population densities are approximately zero.

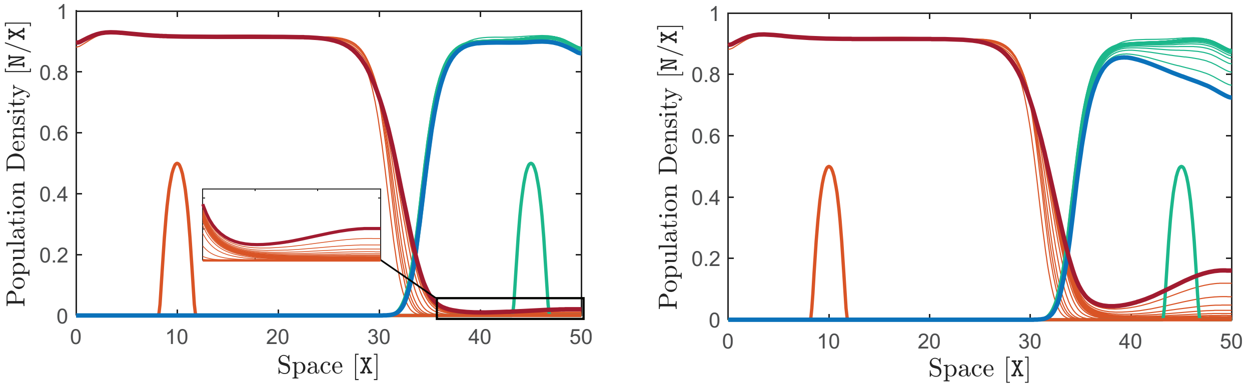

(a) Population densities up to $t = 860$ T

(b) Population densities up to $t = 1100$ T

Fig H: Disruption of range limits stability in the absence of Allee effects. The same simulation as shown in Fig G is performed here but in the absence of Allee effects. Curves of population densities are shown. In (a), the simulation is performed up to the time $t = 860$ T. In (b) the simulation is allowed to continue up to the time $t = 1100$ T. Similar to Fig G, range limits are formed at the wiggle approximately at time $t = 700$ T. For clarity, curves are shown only for the time interval after the formation of the range limits. Due to the continuum nature of the model, the stronger species can gradually expand in infinitesimal density (as small as 10^{-15} N/X, starting from the beginning of the simulation) and leak into the geographic range of the weaker species. In the absence of Allee effect, this infinitesimal population enjoys a high positive growth rate (whereas in the presence of Allee effect this population has a negative growth rate). When simulation is performed for a sufficiently long time, this population grows to a density that becomes visible as a tail in the population density of the stringer species, as shown in (a). This tail keeps growing as the simulation continues for longer time (shown in (b)), and eventually results in extinction or marginal existence of the weaker species.

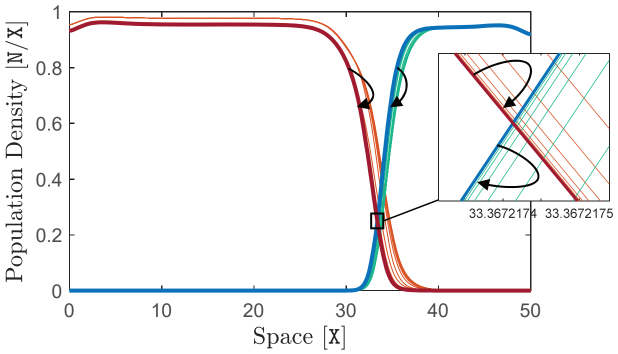

(a) Perturbation in R_1

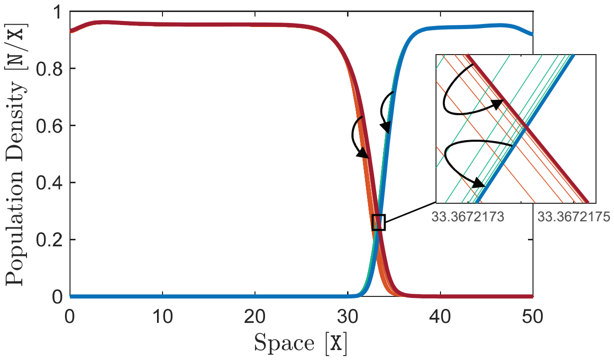

(b) Perturbation in D_1

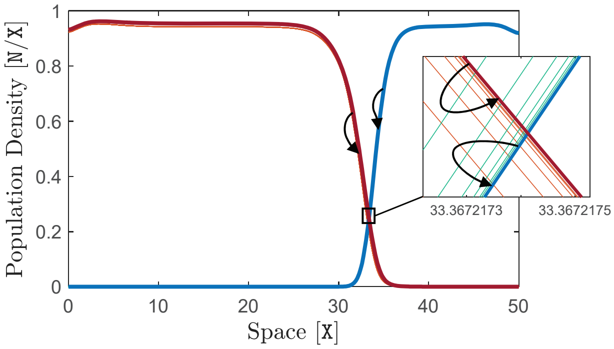

(c) Perturbation in V_1

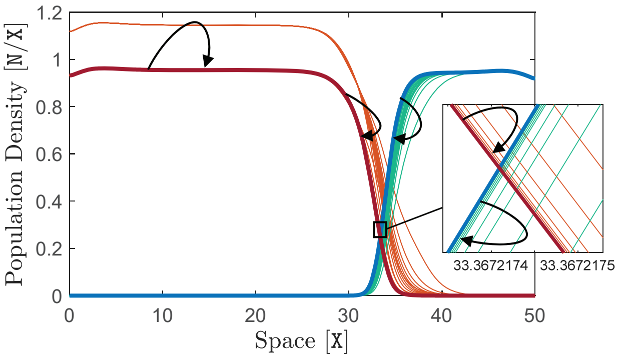

(d) Perturbation in K_1

Fig I: Robustness of the stability of the range limits formed at wiggles against transient perturbations in species' parameters. The two species are initialized at $t = 0$ T by the final solution curves (equilibrium state) obtained at the end of the simulation shown in the lower panel of Fig 3 in the main text (in which the range limits are stabilized at an environmental wiggle). Both species take the same parameter values as used in Fig 3, except for one of the parameters that is perturbed for the downslope species in each panel by an additive rectangular-shaped perturbation of amplitude $\alpha = 20\%$ and duration $t_e = 200$ T; see graph (*iii*) in Fig 8 in Box 2 of the main text. In (a), R_1 is increased by 20% to the value $R_1 = 1.32$ T at the beginning of the simulation, is kept constant at this value for a period of 200 T, and then is decreased back to its initial value $R_1 = 1.1$ T. Similarly, in (b), D_1 is increased by 20% to $D_1 = 1.20$ X^2/T, and then is decreased back to its initial value $D_1 = 1$ X^2/T after 200 T. In (c), V_1 is perturbed by 20% over a time period of 200 T. In (d), a similar perturbation is applied to K_1. In each case, the simulation is performed for a time horizon of $T = 1500$ T. Curves of population density are shown in each graph at every 20 T, and the final curves at $t = 1500$ T are highlighted. The same description as given in Fig 2 of the main text holds for the curve colors and arrows. In all cases, we observe that the stability of the range limits formed at the wiggle is maintained under moderately strong transient perturbations that make the downslope (stronger) species temporarily stronger (in (a) and (d)) or weaker (in (b) and (c)). In particular, the initial equilibrium range limits are precisely restored at the end of the simulation, as the perturbations only occur transiently for a duration of 200 T at the beginning of the simulation.

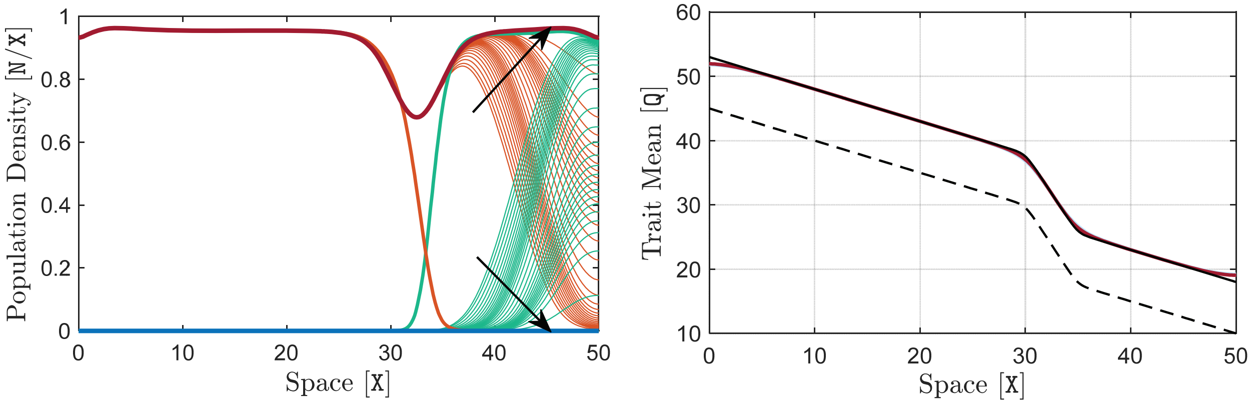

(a) Population densities

(b) Trait means

Fig J: Destabilization of the range limits by a strong climate-warming disturbance when the downslope species is stronger, leading to the exclusion of the upslope species. The same simulation as presented in Fig 6 of the main text is further continued here until a longer evolution time of $T = 2000$ `T`. Curves of population density are shown in (a) at every 50 `T`. The final curves at $t = 2000$ `T` are highlighted in red and blue, and their corresponding trait mean curves are shown in (b). The same description as given in Fig 5 of the main text holds for curve colors, the arrow, and the dashed curve. Note that the final curve of trait mean for the upslope species is made entirely transparent since the final population density of this species is equal to zero everywhere in the habitat and hence its trait mean is not biologically meaningful.

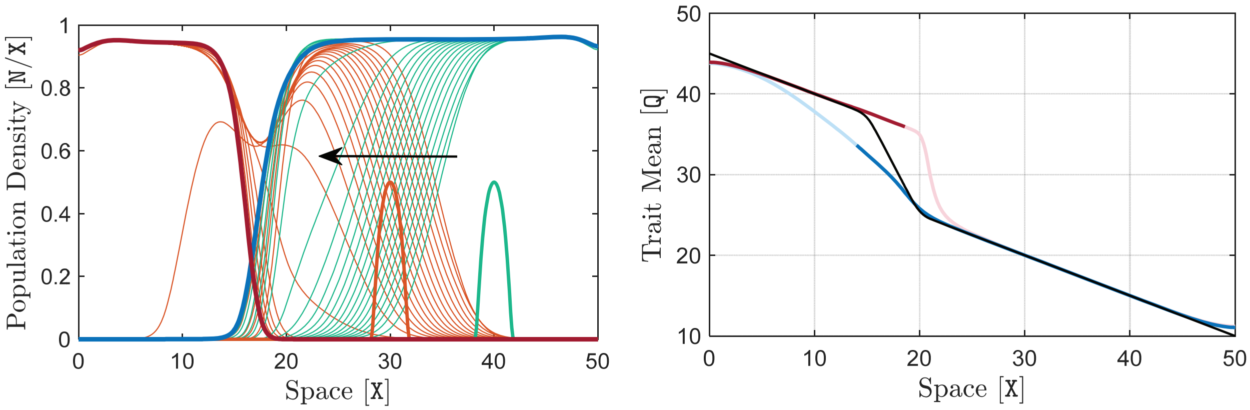

(a) Population densities

(b) Trait means

Fig K: Formation of stable range limits at an environmental wiggle when the upslope species is stronger. A similar simulation to the one presented in the lower panel of Fig 3 in the main text is performed here, with the main difference being that here the upslope species is made stronger. That is, we set $\mathrm{R}_1 = 1\ \mathtt{T}^{-1}$ and $\mathrm{R}_2 = 1.1\ \mathtt{T}^{-1}$. The rest of the parameters take their typical values given in Table 1 of the main text. The simulation is performed for a time horizon of $T = 1500\ \mathtt{T}$ and curves of population density are shown in (a) at every 30 $\mathtt{T}$. The same description as given in Fig 2 of the main text holds for the curve colors and the arrow. The curves highlighted in red and blue are associated with the equilibrium state of the populations, reached (approximately) at the end of the simulation ($t = 1500\ \mathtt{T}$). The highlighted equilibrium curves in (a) represent the evolutionarily stable range limits formed at the environmental wiggle, with their corresponding curves of trait mean shown in (b). The black line in (b) shows the environmental trait optimum Q, whose slope switches sharply from $-0.5\ \mathtt{Q}/\mathtt{X}$ to $-2.5\ \mathtt{Q}/\mathtt{X}$ between the two knees of the wiggle located at $x = 15\ \mathtt{X}$ and $x = 20\ \mathtt{X}$. The curves of trait mean in (b) are made transparent over the regions where population densities are approximately zero, as the values of trait mean over these regions are not biologically meaningful.

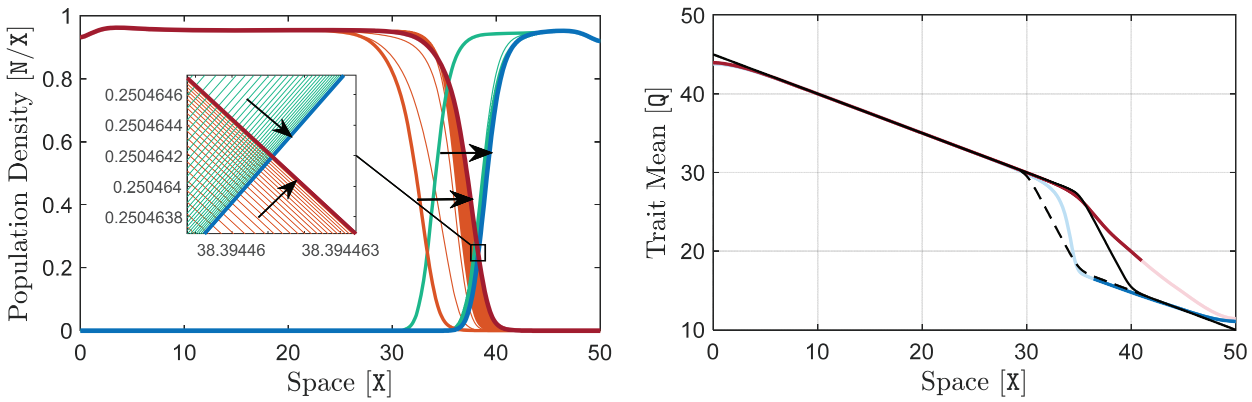

(a) Population densities **(b)** Trait means

Fig L: Range shifts resulting from an environmental disturbance that moves the wiggle towards the weaker species. The simulation is performed using the same model parameters as used in Fig 3 of the main text. The equilibrium curves obtained at the end ($t = 1500$ T) of the simulation shown in Fig 3 of the main text are used as the initial curves here. At $t = 0$ T, the wiggle in the trait optimum Q is immediately moved upslope by 5 X and remains unchanged afterwards. The initial curve of Q is shown by the dashed black line in (b), and the shifted curve after $t = 0$ T is shown by the solid black line. The simulation is performed for a time horizon of $T = 1500$ T. Curves of population density are shown at every 2 T in (a), and the final curves obtained at $t = 1500$ T are highlighted. The final curves of trait mean obtained at $t = 1500$ T are shown in (b). The same description as given in Fig 2 of the main text holds for the curve colors. We observe that the range limits move and get stabilized at the new location of the wiggle.

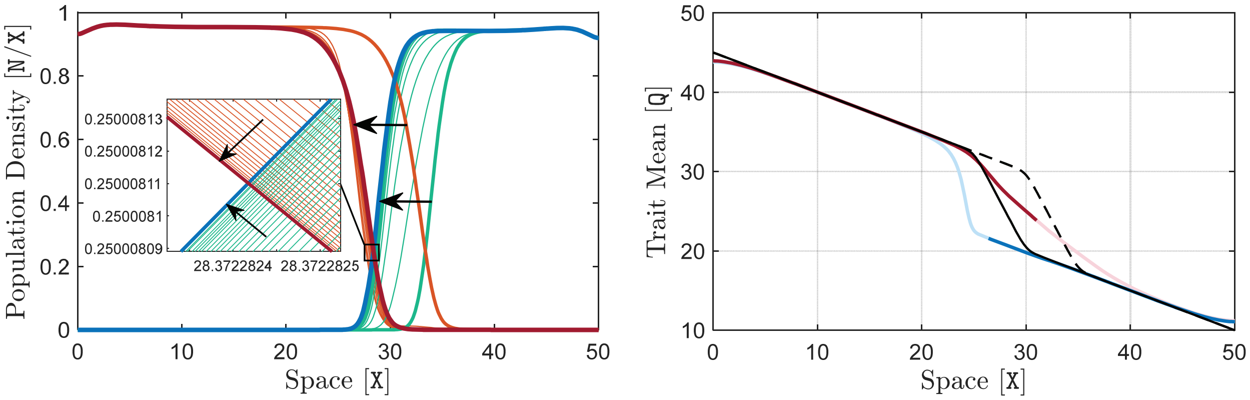

(a) Population densities

(b) Trait means

Fig M: Range shifts resulting from an environmental disturbance that moves the wiggle towards the stronger species. The same simulation as in Fig L is performed here, but with a disturbance that moves the wiggle downslope by 5 X. Since the disturbance is sufficiently small (even though it is a relatively large disturbance), we observe that the range limits move and get stabilized at the new location of the wiggle.
