# Supplementary material for: Environmental “knees” and “wiggles” as strong stabilizers of species’ range limits set by interspecific competition
Source: PLoS Comput Biol. 2026 Jun 15;22(6):e1014336. doi: 10.1371/journal.pcbi.1014336 (PMC13278590; doi:10.1371/journal.pcbi.1014336)
Supplement: S1 Appendix — This file includes all appendices referenced in the main text. (PDF) [file pcbi.1014336.s002.pdf]

Environmental “Knees” and “Wiggles” as Strong Stabilizers of Species’ Range Limits Set by Interspecific Competition (Appendix)

Farshad Shirani[*,†,‡]   Benjamin G. Freeman[§]

Contents

A Notational Differences with Preceding Models **2**

B Assumption of Normal Distribution of Phenotypes **3**

C Numerical Methods **4**

D Range Expansion Dynamics and the Effects of Random Gene Flow **5**

[*]School of Mathematics, Georgia Institute of Technology, Atlanta, GA 30332, USA
[†]Department of Physics, Emory University, Atlanta, GA 30322, USA
[‡]Department of Mathematics and Statistics, Georgetown University, Washington, DC 20057, USA
[§]School of Biological Sciences, Georgia Institute of Technology, Atlanta, GA 30332, USA

A. Notational Differences with Preceding Models

In presenting the equations and the underlying components of the model we used the same mathematical notations as in [1,2]. To distinguish model variables from model parameters and mappings of model variables, to avoid conflicts with some commonly used notations in mathematical analysis of PDEs, and to allow for additional notational styles for possible future extensions and analyses of the model, some notational changes have been made in [1,2], compared with the notations used in the foundational models of Kirkpatrick & Barton [3] and Case & Taper [4]. To facilitate comparisons with the preceding models, in Table 2 of the main text we provide the list of model parameters and variables, with the notations used in the present work (as well as in [1,2]) and those used in [4] (as well as in [3] and other works in this family of models). Below, we also rewrite the key equations of the model (presented in th main text) using the notation used by Case and Taper. Since trait variance is free to evolve in our model, we still use our notation v_i, $i = 1, 2$, to denote species' trait variance (instead of the notation V_p used in Case and Taper's constant-variance model).

The master equation (1) in the main text, representing the change in $N_i(x,t)p_i(x,t,z)$ over a small time interval of length $\tau \to 0$ is given as

$$\begin{aligned} N_i(x,t+\tau)p_i(x,t+\tau,z) - {}& N_i(x,t)p_i(x,t,z) \\ &= \tau D_i\,\partial_x^2\big(N_i(x,t)p_i(x,t,z)\big) && \text{(A1a)} \\ &\quad + \tau w_i(x,t,z)N_i(x,t)p_i(x,t,z) && \text{(A1b)} \\ &\quad + \tau N_i(x,t)\partial_t^{(\mathrm{M})}p_i(x,t,z). && \text{(A1c)} \end{aligned}$$

The intrinsic growth rate (Eq. (2) of the main text) is written as

$$\begin{aligned} w_i(x,t,z) := r_i &\left(1 - \frac{1}{K_i}\sum_{j=1}^{2} N_j(x,t)\int_{\mathbb{R}} \alpha_{ij}(z,z')p_j(x,t,z')\mathrm{d}z'\right) B_i(N_i(x,t)) && \text{(A2a)} \\ &- \frac{\big(z-\theta(x)\big)^2}{2V_{\mathrm{s}}}, && \text{(A2b)} \end{aligned}$$

where the competition kernel (Eq. (3) of the main text) is given as

$$\alpha_{ij}(z,z') = \sqrt{V_{\mathrm{u}i}/\bar{V}_{\mathrm{u}ij}}\,\exp\left(-\frac{(z-z')^2}{4\bar{V}_{\mathrm{u}ij}}\right), \quad i,j \in \{1,2\}, \tag{A3}$$

with $\bar{V}_{\mathrm{u}ij} := \frac{1}{2}(V_{\mathrm{u}i} + V_{\mathrm{u}j})$. The nonlinear function B_i that incorporates Allee effect to the model (Eq. (4) of th emain text) is as follows.

$$B(N_i) := \mathrm{B}_i\left(\frac{1}{1+\exp\left(-\frac{N_i - \mathrm{J}_i}{\sigma_i}\right)} - \frac{1}{2}\right), \tag{A4}$$

The equation for the evolution of the population density (Eq. (5) of the main text) is given by

$$\partial_t N_i(x,t) = D_i\partial_x^2 N_i(x,t) + \bar{w}(x,u(x,t))N_i(x,t), \tag{A5}$$

the equations for the evolution of the trait mean (Eq. (6) of the main text) is given by

$$\partial_t \bar{z}_i(x,t) = D_i\partial_x^2\bar{z}_i(x,t) + 2\,D_i\partial_x\big(\log N_i(x,t)\big)\partial_x\bar{z}_i(x,t) + H_i(x,u(x,t)), \tag{A6}$$

and the equations for the evolution of the trait variance (Eq. (7) of the main text) is given by

$$\begin{aligned}\partial_t v_i(x,t) = D_i \partial_x^2 v_i(x,t) + 2\,D_i \partial_x\big(\log N_i(x,t)\big)\partial_x v_i(x,t) \\ + 2\,D_i\big(\partial_x \bar{z}_i(x,t)\big)^2 + W_i(x,u(x,t)).\end{aligned} \tag{A7}$$

The nonlinear terms $\bar{w}_i$, H_i, and W_i used in (A5)–(A7) are defined as

$$\bar{w}_i(x,u) = B_i(N_i)\left(r_i - \frac{r_i}{K_i}\sum_{j=1}^{2} M_{ij}(u)C_{ij}(u)N_j\right) - \frac{\big(\bar{z}_i - \theta(x)\big)^2 + v_i}{2V\mathrm{s}}, \tag{A8}$$

$$H_i(x,u) = \Big(B_i(N_i)r_i - \bar{w}_i(x,u)\Big)\bar{z}_i - B_i(N_i)\frac{r_i}{K_i}\sum_{j=1}^{2} L_{ij}(u)M_{ij}(u)C_{ij}(u)N_j + E_i(x,u), \tag{A9}$$

$$W_i(x,u) = \Big(B_i(N_i)r_i - \bar{w}_i(x,u)\Big)(v_i - \bar{z}_i^2) - B_i(N_i)\frac{r_i}{K_i}\sum_{j=1}^{2} P_{ij}(u)M_{ij}(u)C_{ij}(u)N_j + Y_i(x,u), \tag{A10}$$

where, letting $\bar{V}_{\mathrm{u}ij} := \frac{1}{2}(V_{\mathrm{u}i} + V_{\mathrm{u}j})$, $i,j \in \{1,2\}$,

$$C_{ij}(u) := \sqrt{\frac{2V_{\mathrm{u}i}}{v_i + v_j + 2\bar{V}_{\mathrm{u}ij}}}, \tag{A11}$$

$$M_{ij}(u) := \exp\left(-\frac{(\bar{z}_i - \bar{z}_j)^2}{2(v_i + v_j + 2\bar{V}_{\mathrm{u}ij})}\right), \tag{A12}$$

$$L_{ij}(u) := \frac{v_i\bar{z}_j + (v_j + 2\bar{V}_{\mathrm{u}ij})\bar{z}_i}{v_i + v_j + 2\bar{V}_{\mathrm{u}ij}}, \tag{A13}$$

$$P_{ij}(u) := \frac{v_i(v_j + 2\bar{V}_{\mathrm{u}ij})}{v_i + v_j + 2\bar{V}_{\mathrm{u}ij}} + L_{ij}(u)(L_{ij}(u) - 2\bar{z}_i), \tag{A14}$$

$$E_i(x,u) := \frac{1}{2V_{\mathrm{s}}}\left[2\theta(x)v_i + 2\theta(x)\bar{z}_i^2 - \theta^2(x)\bar{z}_i - 3v_i\bar{z}_i - \bar{z}_i^3\right], \tag{A15}$$

$$Y_i(x,u) := \frac{1}{2V_{\mathrm{s}}}\left[2\theta(x)v_i\bar{z}_i - 2\theta(x)\bar{z}_i^3 - \theta^2(x)(v_i - \bar{z}_i^2) - 3v_i^2 + \bar{z}_i^4\right] + \mathrm{U}, \tag{A16}$$

which correspond, respectively, to Eqs. (11)–(16) in the main text.

B. Assumption of Normal Distribution of Phenotypes

The assumption that the distribution of phenotypes is normal is a relatively strong assumption, made for the simplicity of model derivation in [1, 3, 4]. However, we do not expect the general results of our work to be significantly affected by the violation of this assumption, as long as the distribution of phenotypes remains to be approximately unimodal and symmetric (not heavily skewed) with finite moments. Within the framework of our study, such conditions are reasonably satisfied. In [4], the normality assumption is made following the assumptions that the trait is determined by many loci, allelic effects are additive within and between loci, individuals mate randomly, and selection is weak.

When the trait optimum changes linearly in space, gene flow is localized (e.g., diffusive), and the majority of genetic variation in the population is maintained by gene flow (migration) rather than

by mutation, it is argued by Barton [5,6] that continuum allelic effects at each loci can be reasonably assumed to be normal. If the allelic effects vary independently, then their sum (additive trait) will also be normally distributed. Knowing that the Central Limit Theorem extends (under certain conditions on the moments) to non-identically distributed random variables, the trait distribution will still tend to be normal if allelic effects are weakly dependent. The analysis performed by [1], as well as the single-species simulations we perform in the present work, confirm that the local phenotypic/genetic variation is determined predominantly by gene flow provided the species' range evolves in sufficiently steep environmental gradients. This is a condition that holds in all of the simulations we perform in this work. Further, the nonlinearities that we study are in fact piecewise linear, with the nonlinear regions only occurring locally at the location of the knees. As we showed, these locations will be occupied by peripheral (low-density) populations of the species and do not contribute significantly to the gene flow across the core of the populations. Therefore, following Barton's arguments, the normality assumption on the distribution of phenotypes should be fairly reasonable in our results, except possibly at the location of the knees. We also note that, the normality of allelic effects at each locus is indeed a much stronger condition than the normality of the overall distribution of phenotype values [6]. That is, the phenotypes can still be normally distributed even when the conditions we described above (high gene flow) is not satisfied.

C. Numerical Methods

In all simulation results presented in this work, the numerical solutions of the model equations are computed using the same numerical method as used in [1], with a slight modification regarding the terms $\partial_x \log n_i$ as described below. The method is based on an implicit scheme with two stabilizing correction stages [7]. In each iteration of the scheme, instead of solving the nonlinear algebraic equations involved in the computations, the linearized version of these equations are solved. The iteration time steps are then made smaller to compensate for the linearization error. The first and second space derivatives are approximated using fourth-order centered differences. See Appendix B of the work by [1] for further details. In all simulations, we discretized the one-dimensional space (habitat) with a uniform mesh of size $\Delta x = 0.1$ X. For the majority of the simulations in which we aimed to compute the equilibrium curves accurately, we used small time steps of length $\Delta t = 0.002$ T. For simulations that required an exceedingly long evolutionary time horizon T, or did not require computation of fully accurate solutions, we chose longer time steps of $\Delta t = 0.005$ T, $\Delta t = 0.01$ T, or $\Delta t = 0.016$ T.

The presence of the terms $\partial_x \log n_i$ in the equations of the model, (A6) and (A7), make the numerical computations of the solutions particularly challenging. These terms are undefined when the population densities n_i are zero; see further details in [1, Section 4.1]. Even if we initialize the populations such that at least an infinitesimal population density is initially present everywhere, for example as $n_i = \operatorname{sech}(|x|)$, there is still a chance of reaching numerical singularities if the density of a species undergoes a long density decline (or extinction) regime, especially with our addition of the Allee effect to the model. To avoid this singularity problem, in our numerical computations we replace the terms $\partial_x \log n_i$ with $\partial_x \log(n_i + \epsilon)$, $\epsilon > 0$. A small value of ϵ, as small as 10^{-5}, works well in our simulations and does not result in any noticeable difference in the solutions of the model compared with the original equations. With this minor modification, we could also initialize the

population densities using bump functions, which are exactly equal to zero outside the support of the functions. We used the bump function

$$\varrho(y) := \begin{cases} \exp\left(1 - \dfrac{1}{1-y^2}\right), & |y| < 1, \\ 0, & |y| \geq 1, \end{cases}$$

which has the compact support $[-1, 1]$ and takes the maximum value 1 at $y = 0$.

D. Range Expansion Dynamics and the Effects of Random Gene Flow

In this section we analyze the effects of gene flow on adaptation and range expansion dynamics of a solitary species. The equations of the model for a single species with symmetric intraspecific competition between phenotypes can be written as

$$\partial_t n = \mathrm{D}\partial_x^2 n + \left(B(n)\mathrm{R} - \frac{B(n)\mathrm{R}}{\mathrm{K}}\sqrt{\frac{\mathrm{V}}{v+\mathrm{V}}}n - \frac{\mathrm{S}}{2}\left[(q-\mathrm{Q})^2 + v\right]\right)n, \tag{A17}$$

$$\partial_t q = \mathrm{D}\partial_x^2 q + 2\mathrm{D}\partial_x(\log n)\partial_x q - \mathrm{S}(q-\mathrm{Q})v, \tag{A18}$$

$$\partial_t v = \mathrm{D}\partial_x^2 v + 2\mathrm{D}\partial_x(\log n)\partial_x v + 2\mathrm{D}|\partial_x q|^2 + \frac{\mathrm{R}}{\mathrm{K}}\sqrt{\frac{\mathrm{V}}{v+\mathrm{V}}}\frac{nv^2}{2(v+\mathrm{V})} - \mathrm{S}v^2 + \mathrm{U}. \tag{A19}$$

Since there exists only one species in the model, the numeration index $i = 1$ of the variables and parameters in (A17)–(A19) is dropped for notational simplicity. Further, the dependence of n, q, and v on x and t, as well as the dependence of Q on x, are not shown for the simplicity of exposition.

As the initially introduced population establishes itself in the habitat, it gradually adapts to new environments under the force of natural selection (Fig A1b) and expands its range in the form of traveling waves (Fig A1a). At the core of the population, the density of the population grows to the environment's maximum capacity and its trait mean converges to the environmental trait optimum. The population's density declines near the edge of its range, which creates an asymmetry in gene flow from the populous core of the population to its edge. Due to the presence of a gradient in trait optimum, the phenotypes that are adapted to central regions of the range will be maladapted at peripheral regions. Therefore, the asymmetric gene flow decreases the mean fitness of the population at its range margins. As a result, population density decreases further near the range edge, which then results in a further increase in the asymmetry of gene flow. This positive feedback loop between population density and gene flow slows down the range expansion of the population. The steeper the environmental gradient, the stronger the maladaptive effects of gene flow and the slower the range expansion speed; see the curves of range expansion speed in [1, Fig. 4] and [2, Fig. 2] for a similar simulation.

To better illustrate the maladaptive effects of random dispersal, the direct effects of dispersal on rate of change of trait mean $\partial_t q$ is shown in Fig A1d, computed using the term $\mathrm{D}\partial_x^2 q + 2\mathrm{D}\partial_x(\log n)\partial_x q$ in (A18). The curves in Fig A1d are shown for the right-half of the species's range, where its trait mean initially lies above the trait optimum at $t = 0\ \mathtt{T}$. Therefore, adaptation occurs when the trait mean decreases to the trait optimum, that is when $\partial_t q$ takes negative values. Positive values of

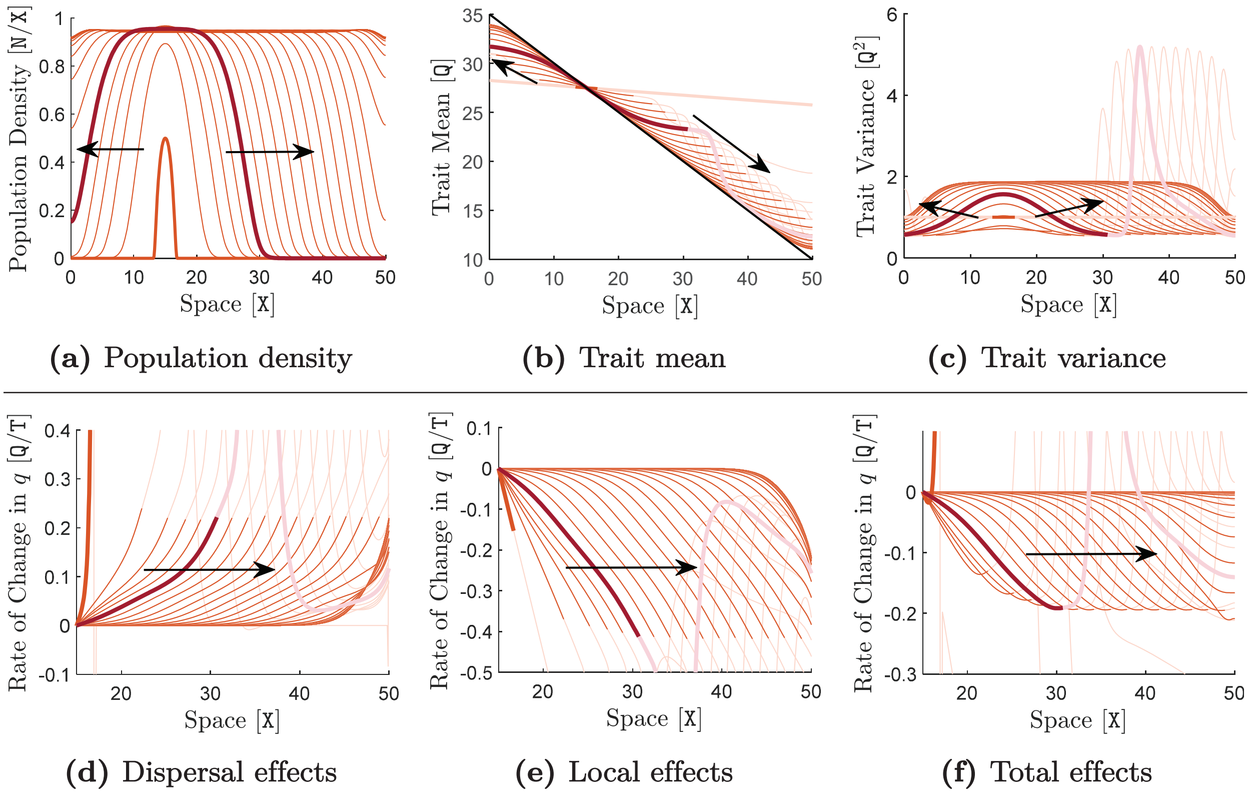

Fig A1: Range evolution of a solitary species and the effects of random gene flow, selection, and competition on population adaptation. The parameters of the species are set equal to the default values given in Table 1 of the main text. The trait optimum Q, shown by the black line in (b), changes linearly with a moderately steep gradient (slope) of $\partial_x \mathrm{Q} = -0.5\ \mathtt{Q}/\mathtt{X}$. Curves of the species' population density, as its range evolves in time, are shown in (a). The corresponding curves of species' trait mean and trait variance are shown in (b) and (c), respectively. The contributions of dispersal (gene flow) as well as the local contributions of selection and competition to the rate of change of trait mean $\partial_t q$ is shown in (d) and (e), respectively. The curves are shown only for the right-half of the species range $(15\ \mathtt{X}, 50\ \mathtt{X})$. Curves in (d) are computed using the term $\mathrm{D}\partial_x^2 q + 2\mathrm{D}\partial_x(\log n)\partial_x q$ in (A18), and curves in (e) are computed using the term $-\mathrm{S}(q-\mathrm{Q})v$. The total contribution to $\partial_t q$, shown in (f), is computed as the sum of these two terms, that is, the sun of the curves shown in (d) and (e). In all graphs, curves are shown at every $4\ \mathtt{T}$ for a simulation time horizon of $T = 200\,\mathtt{T}$. The parts of the curves which lie outside the species' range $(n < 0.02)$ are made transparent, as the values of trait mean and trait variance over these regions are not biologically meaningful. The thick orange curves indicate the initial curves at $t = 0\,\mathtt{T}$, and arrows show the direction of evolution in time. In each graph, a sample curve at $t = 20\,\mathtt{T}$ is highlighted in red.

$\partial_t q$ will then imply maladaptive effects. As we see in Fig A1d, the effects of random dispersal that brings phenotypes from the core to the edge are maladaptive at range margins.

The gene flow along the gradient will, on the other hand, increase genetic variation in local populations; the steeper the gradient the higher the level of genetic variation [2, Fig 2a]. In the models that assume constant phenotypic variance, the swamping effects of asymmetric gene flow on

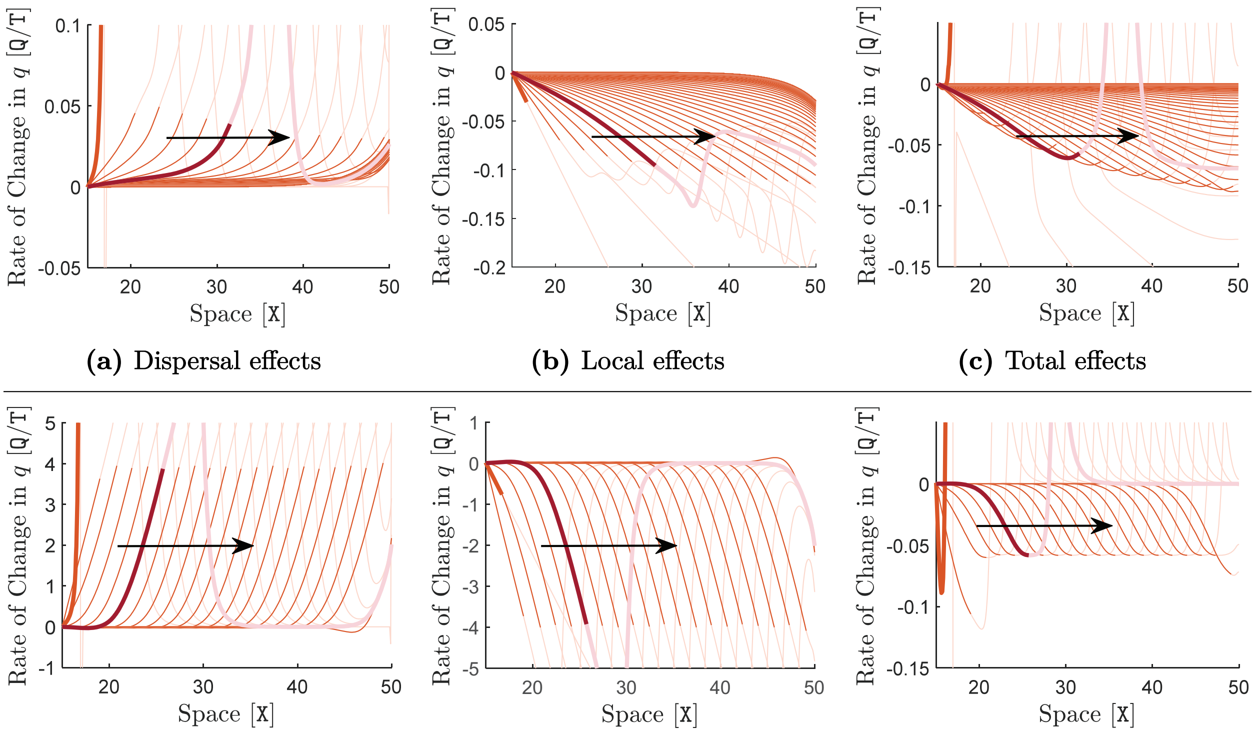

(a) Dispersal effects **(b)** Local effects **(c)** Total effects

(d) Dispersal effects **(e)** Local effects **(f)** Total effects

Fig A2: Effects of random gene flow and selection on population adaptation in shallow and steep environmental gradients. The same simulation as in Fig 1 of the main text is performed here, but with a shallow gradient of $\partial_x \mathrm{Q} = -0.1$ $\mathtt{Q/X}$ for the graphs shown in the upper panel, and a steep gradient of $\partial_x \mathrm{Q} = -2.5$ $\mathtt{Q/X}$ for the graphs shown in the lower panel. The contributions of dispersal (gene flow) to the rate of change of trait mean $\partial_t q$ are shown in (a) and (d), the local contributions of selection and competitions are shown in (b) and (e), and total contribution are shown in (c) and (f). These contributions are computed in the same way as described in Fig 1 of the main text. The curves are shown only for the right-half of the species range ($15\,\mathtt{X}, 50\,\mathtt{X}$). The curves in the upper panel (shallow gradient) are shown at every $2\ \mathtt{T}$ for a simulation time horizon of $T = 200\,\mathtt{T}$, with a sample curve being highlighted in red at $t = 10\,\mathtt{T}$. The curves in the lower panel (steep gradient) are shown at every $40\ \mathtt{T}$ for a simulation time horizon of $T = 800\,\mathtt{T}$, with a sample curve being highlighted in red at $t = 200\,\mathtt{T}$. The same description as given in Fig 1 of the main text holds for the curve colors and arrows.

adaptation at the range edge will become so strong at steep gradients that range expansion will halt [3]. However, when we allow trait variance to evolve (Fig A1c), the inflation in phenotypic variation caused by gene flow facilitates adaptation by natural selection. This is because increased trait variation provides more opportunities for natural selection to operate, allowing for local adaptation and range expansion even at exceedingly steep gradients. Figs A1d–A1f illustrate the overall mechanism of adaptation at range margins. The term $-\mathrm{S}(q - \mathrm{Q})v$ in (A18), shown in Fig A1e, represents the local adaptation by selection acting on the genetic variation created by gene flow, mutation, and intraspecific competition. We note that in relatively steep gradients such as that simulated in Fig A1, the effects of mutation and intraspecific competition on creating genetic variation are rather insignificant compared with the dominant effects of gene flow. As Figs A1d–

A1f show, the local adaptation caused by selection is strong enough to overcome the maladaptive effects of random dispersal, allowing for adaptive range expansion to new environments. Note that negative values in Figs A1d–A1f imply adaptive effects, because the trait mean on the right-half of the species' range is initially above the trait optimum.

The inflating effects of random gene flow on trait variation and thereby increasing the population's adaptive potential creates ambiguities on our general reference to "maladaptive" effects of gene flow, which we need to clarify. The results shown in Fig A1 were obtained for a moderately steep gradient of $\partial_x \mathrm{Q} = -0.5\ \mathtt{Q/X}$. To clarify the effects of gene flow, we repeat the same simulation but with a much shallower gradient of $\partial_x \mathrm{Q} = -0.1\ \mathtt{Q/X}$ as well as a much steeper gradient of $\partial_x \mathrm{Q} = -2.5\ \mathtt{Q/X}$. The results are shown in Fig A2. Comparing Figs A1d, A2a, and A2d confirms that the disruptive effects of core-to-edge gene flow, which deviate the trait mean from trait optimum, increase as the level of gene flow increases with steepness of the environment. In contrast, Figs A1e, A2b, and A2e confirm that the adaptive potential of the peripheral population increases with the steepness of the environment. This is because trait variation increases as the population disperses and adapts along steeper gradients, mainly due to the term $2\mathrm{D}|\partial_x q|^2$ in (A19). The sum of these two contrasting effects gives the total rate of change in trait mean $\partial_t q$, shown in Figs A1f, A2c and A2f, which changes in a more complicated way with increases in environmental gradient.

Noting that more negative values of $\partial_t q$ in the results presented here imply faster adaptation, Figs A1f, A2c and A2f show that adaptation rate at range margins is higher when the environment is moderately steep. This could imply that the overall effects of intermediate levels of gene flow is adaptive at range margins, and hence could facilitate range expansion compared with low or very high levels of gene flow. However, examining the range expansion speeds in Figs A1 and A2, as well as the speeds shown in [1, Fig. 4] and [2, Fig. 2a] contradicts this conjecture. Range expansion speed decreases monotonically as the steepness of the environmental gradient and hence the level of gene flow increases. This means that, even though the maladapted phenotypes that are brought to the range periphery from the range core also create an adaptive potential to mitigate their maladaptive effects, the migration load they impose on the population fitness (deviation of the trait mean from the trait optimum [8]) is always too strong to be fully compensated by the effects of increased adaptive potential. To further elaborate on this, we repeat the simulation associated with Fig A1 but with an initial population that is perfectly adapted to the trait optimum, $q = \mathrm{Q}$ at $t = 0\ \mathtt{T}$. The results are shown in Fig A3. If the overall effects of gene flow were adaptive, this initial population would remain perfectly adapted for all time. Instead, we see that gene flow quickly deviates the trait mean of the peripheral population from the optimum, and adaptation and range expansion proceeds almost the same as in Fig A1.

It is worth pointing out that the overall adaptation at range expansion wavefronts, as in Figs A1f, A2c and A2f, does not imply that the species will be able to expand its range in arbitrarily steep gradients. Natural selection imposes a phenotypic load on the mean growth rate of the population, given by the term $-\frac{\mathrm{S}}{2}v$ in (A17), which increases proportionally with the inflation in trait variance as the environmental gradient becomes steeper. At exceedingly steep gradients, the phenotypic load becomes so strong that brings the population to extinction. In the absence of Allee effect, a maximum steepness of the gradient in trait optimum is given in [1] as $|\partial_x \mathrm{Q}|_{\max} = \sqrt{(2\mathrm{R}^2/\mathrm{SD}) - \mathrm{U}/2\mathrm{D}}$, beyond which the population cannot survive—unless possibly marginally at the vicinity of the habitat boundary. This estimate of the critical (extinction) gradient steepness is

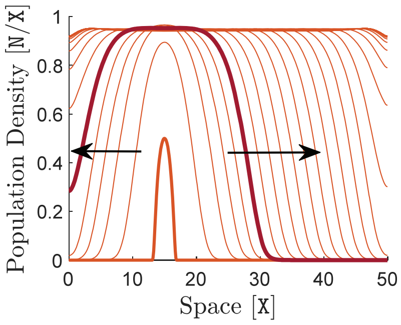

(a) Population density

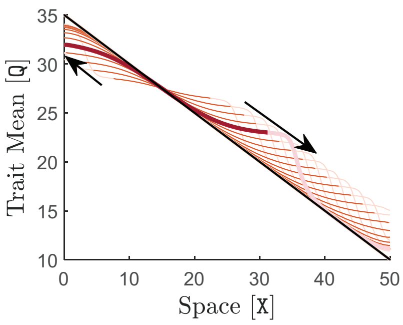

(b) Trait mean

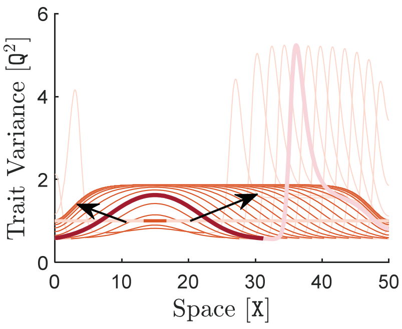

(c) Trait variance

Fig A3: Range evolution of a solitary species with perfect initial adaptation. The same simulation as in Fig 1 of the main text is performed here, with the only difference being that the population is initialized to be perfectly adapted to the environment, that is, $q(x,0) = \mathrm{Q}(x)$ for all $x \in \Omega$. The same descriptions as given in Figs 1a–1c of the main text hold for the quantities shown in the graphs, curve colors, arrows, time increments between the curves, and the simulation time horizon.

still approximately valid after our addition of the Allee effect to the model. Note that, the results in [1, Fig. 4] and [2, Fig. 2a] show that the range expansion speed also vanishes to zero exactly at this critical gradient, and remains nonzero at shallower gradients. This further implies that in linearly changing environments, genetic swamping cannot establish range limits when trait variance is free to evolve [1,5].

Finally, we note that, throughout the present work, by maladaptive (swamping) effects of gene flow we mean the load that migration imposes on peripheral populations' fitness by bringing phenotypes that are adapted to the abundant core but maladapted at the edge, hence deviating the peripheral populations' trait mean from the trait optimum. That is, the maladaptive effects shown in Figs A1d, A2a and A2d.

References

[1] Farshad Shirani and Judith R. Miller. Competition, trait variance dynamics, and the evolution of a species' range. *Bulletin of Mathematical Biology*, 84(3):37, 2022.

[2] Farshad Shirani and Judith R. Miller. Matching habitat choice and the evolution of a species' range. *Bulletin of Mathematical Biology*, 87(6):1–57, 2025.

[3] M. Kirkpatrick and N. H. Barton. Evolution of a species' range. *The American Naturalist*, 150(1):1–23, 1997.

[4] T. J. Case and M. L. Taper. Interspecific competition, environmental gradients, gene flow, and the coevolution of species' borders. *The American Naturalist*, 155(5):583–605, 2000.

[5] N. Barton. Adaptation at the edge of a species range. In J. Silvertown and J. Antonovics, editors, *Integrating ecology and evolution in a spatial context*, chapter 17, page 365–392. Blackwell, Oxford, 2001.

[6] N. H. Barton. Clines in polygenic traits. *Genetical Research*, 74(3):223–236, 1999.

[7] W. Hundsdorfer. Accuracy and stability of splitting with stabilizing corrections. *Applied Numerical Mathematics*, 42(1):213–233, 2002.

[8] Thomas Lenormand. Gene flow and the limits to natural selection. *Trends in Ecology & Evolution*, 17(4):183–189, 2002.
